# Insights into the transcriptional and post-transcriptional regulation of the rice SUMOylation machinery and into the role of two rice SUMO proteases

**DOI:** 10.1186/s12870-018-1547-3

**Published:** 2018-12-12

**Authors:** Margarida T. G. Rosa, Diego M. Almeida, Inês S. Pires, Daniel da Rosa Farias, Alice G. Martins, Luciano Carlos da Maia, António Costa de Oliveira, Nelson J. M. Saibo, M. Margarida Oliveira, Isabel A. Abreu

**Affiliations:** 1 Instituto de Tecnologia Química e Biológica António Xavier, Universidade Nova de Lisboa (ITQB-UNL), Av. da República, 2780-157 Oeiras, Portugal; 2 IBET, Av. da República, 2780-157 Oeiras, Portugal; 3 Plant Genomics and Breeding Center, Faculdade de Agronomia Eliseu Maciel, Universidade Federal de Pelotas, Pelotas, RS Brazil; 4 Laboratoire de Biochimie et Physiologie Moléculaire des Plantes (BPMP), Institut National de la Recherche Agronomique (INRA), Université de Montpellier (UM), Montpellier, France; 5 Frontiers Media SA, Avenue du Tribunal-Fédéral 34, CH-1015 Lausanne, Switzerland

**Keywords:** SUMOylation, *cis*-elements, Rice (*Oryza sativa*), Alternative splicing, T-DNA, SUMO proteases

## Abstract

**Background:**

SUMOylation is an essential eukaryotic post-translation modification that, in plants, regulates numerous cellular processes, ranging from seed development to stress response. Using rice as a model crop plant, we searched for potential regulatory points that may influence the activity of the rice SUMOylation machinery genes.

**Results:**

We analyzed the presence of putative *cis*-acting regulatory elements (CREs) within the promoter regions of the rice SUMOylation machinery genes and found CREs related to different cellular processes, including hormone signaling. We confirmed that the transcript levels of genes involved in target-SUMOylation, containing ABA- and GA-related CREs, are responsive to treatments with these hormones. Transcriptional analysis in Nipponbare (spp. *japonica*) and LC-93-4 (spp. *indica*), showed that the transcript levels of all studied genes are maintained in the two subspecies, under normal growth. *OsSUMO3* is an exceptional case since it is expressed at low levels or is not detectable at all in LC-93-4 roots and shoots, respectively. We revealed post-transcriptional regulation by alternative splicing (AS) for all genes studied, except for SUMO coding genes, *OsSIZ2*, *OsOTS3*, and *OsELS2*. Some AS forms have the potential to alter protein domains and catalytic centers. We also performed the molecular and phenotypic characterization of T-DNA insertion lines of some of the genes under study. Knockouts of *OsFUG1* and *OsELS1* showed increased SUMOylation levels and non-overlapping phenotypes. The *fug1* line showed a dwarf phenotype, and significant defects in fertility, seed weight, and panicle architecture, while the *els1* line showed early flowering and decreased plant height. We suggest that *OsELS1* is an ortholog of *AtEsd4*, which was also supported by our phylogenetic analysis.

**Conclusions:**

Overall, we provide a comprehensive analysis of the rice SUMOylation machinery and discuss possible effects of the regulation of these genes at the transcriptional and post-transcriptional level. We also contribute to the characterization of two rice SUMO proteases, *OsELS1* and *OsFUG1*.

**Electronic supplementary material:**

The online version of this article (10.1186/s12870-018-1547-3) contains supplementary material, which is available to authorized users.

## Background

SUMOylation is an essential post-translational modification (PTM) found in all eukaryotes, controlling numerous cellular processes including cell cycle progression, chromatin structure, DNA repair, transcription, transport, signaling, and stress response [1–3], to name a few. Small Ubiquitin-like Modifier (SUMO) is attached to the target protein by a conjugation system resembling the ubiquitination system [4]. SUMO is synthesized as a precursor and needs to be processed by SUMO proteases (Ulp1-like) to expose its diglycine motif at the C-terminus. E1 SUMO-activating enzyme (SAE), a heterodimer constituted by a regulatory subunit (SAE1) and a catalytic subunit (SAE2), activates the processed SUMO. This process requires ATP for the formation of the thioester bond between the cysteine residue in SAE2 and the glycine in SUMO. Activated SUMO is transferred to the catalytic cysteine residue in the E2 SUMO-conjugating enzyme (SCE/Ubc9). Unlike the ubiquitination machinery, E2 can transfer SUMO directly to the lysine residue in the target protein forming an isopeptide bond. This lysine is usually in a consensus sequence ψKxD/E (ψ, large hydrophobic residue, x any amino acid) [1], although other extended motifs have been found [5]. The conjugation step may be enhanced by E3 SUMO ligases, belonging to two different classes: HIGH PLOIDY2 (HPY2/MMS21) and SAP/MIZ1 (SIZ1) [6, 7]. E4 PIAL proteins mediate the formation of polySUMO chains [8]. SUMO proteases also recycle SUMO from the target protein by cleaving the isopeptide bond. The polySUMO chains are targets of specific proteases called Ulp2 (now known as SPF-family, in plants) [9].

In plants, SUMOylation is crucial for development, hormone signaling, light regulation, flowering time, biotic and abiotic stress responses [2, 4, 10]. Experimental data have determined essential roles for some SUMOylation machinery elements. In Arabidopsis, the knockout of *SAE2*, *SCE1a*, and the double knockouts of *SUMO1/2* and *SIZ1/HPY2* are embryo lethal [1]. Individual knockout lines of each ligase show a strong dwarf phenotype [11], whereas the knockout of both *AtPIAL1/2* shows no influence on phenotype during development [8]. The three classes of ligases identified so far do not seem to have overlapping roles, and HPY2 specifically acts during endoreplication, DNA repair, and the maintenance of root stem cell niche [12–14]. SIZ1 has a broader role and is involved in seed germination [15, 16], growth [6, 17], nutrient metabolism [6, 18–22], response to biotic [23] and abiotic [24–28] stresses, hormone signaling [29–31], and light response [32, 33]. The SUMO proteases family also show specific roles for each member, mainly involved in development [34, 35], flowering time [34, 36] and salt stress [37].

So far, the majority of the knowledge about SUMOylation gathered in plants is due to works based on the model organism *Arabidopsis thaliana*. The comparison of the obtained results with the effects of SUMOylation in rice (*Oryza sativa* L.), a monocot model plant, will help to understand the overall impact of the SUMOylation process. Also, rice is one of the most important staple food crop worldwide, providing 50–80% of the daily human calories intake [38]. As an example of an interesting difference between the Arabidopsis and rice SUMOylation machinery, rice has three genes identified as putative SCEs, contrary to Arabidopsis (or yeast and mammals) where only one SCE encoding-gene is present, making it an essential component for Arabidopsis viability [1, 39, 40]. Interestingly, specific responses by *OsSCE* genes to different abiotic stress conditions have been observed [41–43], and *OsSIZ1* overexpression led to increased resistance to abiotic stress [44, 45]. The rice SUMO proteases OsOTS1/2 have a role in seed germination [46] and in salt and drought responses [47, 48].

As mentioned above, SUMOylation has emerged as a regulator of many cellular processes. Thus, we asked if these processes have the potential to regulate the expression of the genes coding for the SUMOylation machinery and thus activate/deactivate SUMOylation when necessary. Actually, in soybean, the search for *cis-*acting regulatory elements (CREs) suggested transcriptional regulation of the machinery that depends on stress conditions, developmental stages, and hormone signaling [49]. Other processes also condition the transcriptional response. Alternative splicing mechanisms are known to create novel regulatory opportunities that influence transcript behavior in different tissues, stages and the response upon environmental changes [50]. In fact, putative alternative splicing forms of the rice SUMOylation machinery have been identified but remained unexplored [51]. Additionally, it has been recently described that the SUMOylation machinery itself is a target of post-translational modifications, but this is mostly unexplored as well. As an example, in plants, AtSIZ1 is known to be regulated by PTMs such as ubiquitination and SUMOylation [28].

Our goal was to contribute to the understanding of the regulation of SUMOylation in rice by studying the genes coding for the SUMOylation machinery. We also took into consideration that thousands of years of domestication resulted in two rice subspecies, *japonica* and *indica*, with profound diversity at the physiological and molecular levels, and thus included two representative rice genotypes in our study. In fact, differences in the gene transcriptional regulation have been found between both subspecies, including the number of alternative spliced transcripts [52]. We started by asking which processes regulate the transcription of the genes coding for the rice SUMOylation machinery, by analyzing the presence of CREs at their promoters. To address the functional relevance of the most prevalent CREs found, we analyzed the transcriptional behavior of selected genes, upon external stimuli. We then compared the transcript levels of the SUMOylation machinery genes and their putative alternative splicing forms (ASFs) within the two rice subspecies, Nipponbare (spp. *japonica*) and LC-93-4 (spp. *indica*). This allowed us to investigate possible effects of the adaptation in the regulatory process of SUMOylation, by exploring the robustness of the transcript regulation of these genes in vegetative state. Finally, we used the available T-DNA lines to address the role of some of the rice SUMOylation machinery genes.

## Results and discussion

In the present work, we started by performing a search for putative CREs (hereafter mentioned as CREs) in the promoter regions of each SUMOylation-related rice gene. We used PLANTCIS for the identification and to exclude randomly existing CREs. Overall, we found a high functional diversity of CREs distributed amongst all analyzed promoters. We identified CREs related to numerous processes such as hormones, stress, light regulation, nutrient metabolism (mainly sugars), and organ- and cell-specific CREs Additional file 1: Table S1 and Table S2). This observation may indicate that SUMOylation is highly regulated by many different cellular processes and may influence diverse aspects of those processes, which is in line with the pleiotropic effect of the disruption of SUMOylation, as has been demonstrated mostly in Arabidopsis with the use of transgenic plants [53, 54]. We also performed a phylogenetic analysis that included sequences from the eukaryotic models *Saccharomyces cerevisiae* and *Arabidopsis thaliana*, and the monocots maize and barley, to help to organize rice genes in the context of previously described functionality in other species. We have further considered the potential post-transcriptional control by alternative splicing (AS), which is a process known to increase proteome diversity [55–57]. Putative alternative splicing forms (ASFs) for all of these genes have been proposed but remained unstudied. To address the putative function of each ASF, we analyzed the presence or absence of protein domains, potential regulatory sites, and catalytic centers. We then quantified the transcript level of each ASF (whenever possible) in roots and shoots of two different rice subspecies, to account for specificity in the use of each ASF. These results are presented and discussed below.

### Characterization of the rice SUMOylation machinery

#### The three SUMO coding genes in rice

**Fig. 1:**
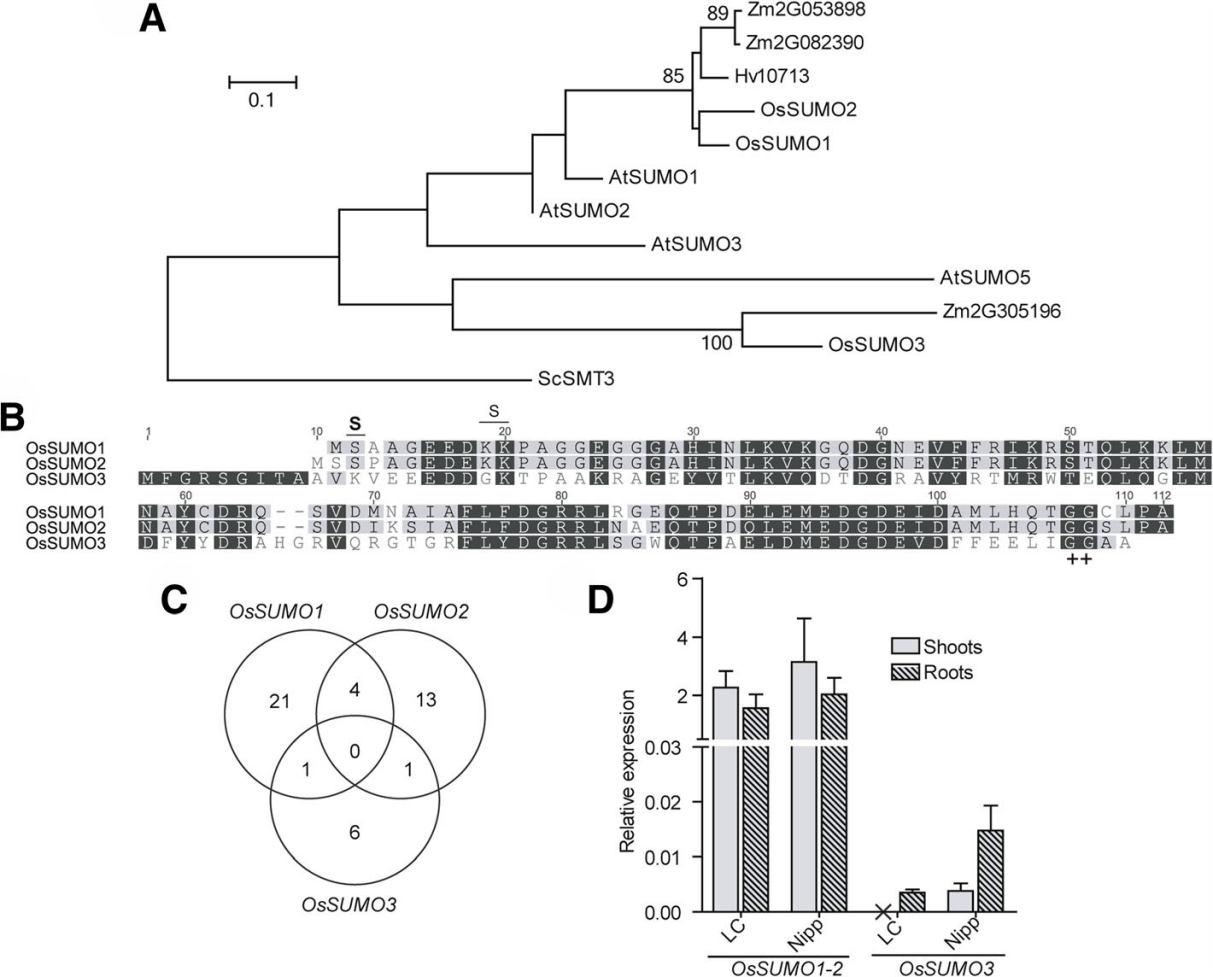
Analyses of three rice putative OsSUMOs proteins. **a** Maximum likelihood phylogeny of the SUMO family. Only nodes with bootstrap support > 75% show the correspondent bootstrap value. Os – *Oryza sativa*, Sc - *Saccharomyces cerevisiae*, At – *Arabidopsis thaliana*, Zm – *Zea mays* and Hv – *Hordeum vulgare*. **b** Protein alignment of the three rice putative SUMO proteins OsSUMO1, OsSUMO2 and OsSUMO3. Diglycine motif is highlighted (++). The lysines with a high probability of being SUMOylated are marked with “S” in bold and the ones with low SUMOylation probability are marked with a regular “S”. **c** Venn diagram showing common CREs in the promoter region of the different *OsSUMOs*. **d** Basal expression levels of *OsSUMO1/2* and *OsSUMO3* genes by qPCR, in shoots (no pattern) and roots (patterned) in LC-93-4 (LC) and Nipponbare (Nipp)

**Fig. 2:**
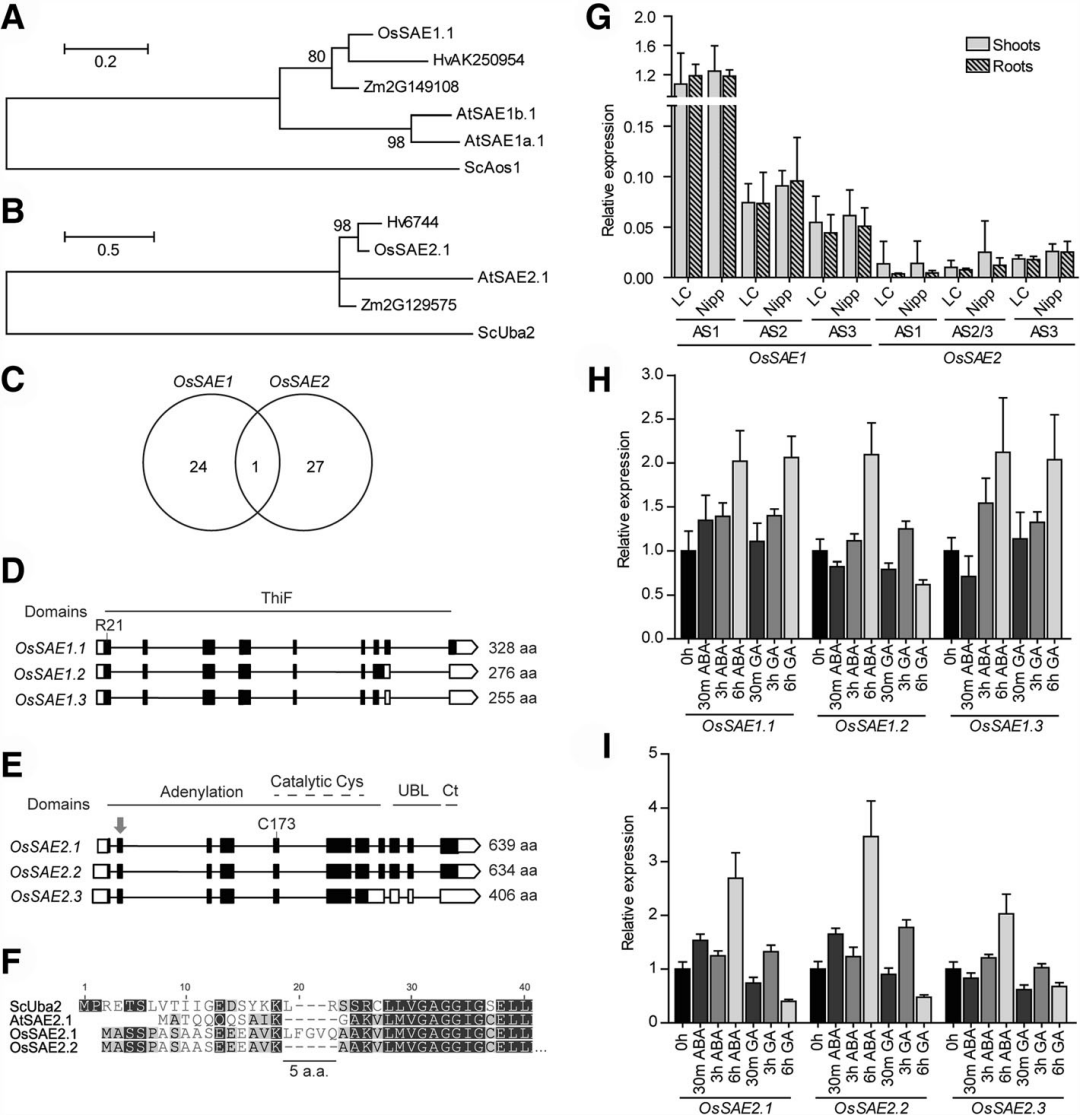
Analysis of the rice genes encoding the SUMO activating enzyme (SAE). The rice E1 is constituted by *OsSAE1* encoding the regulatory subunit and *OsSAE2* encoding the catalytic subunit. Maximum likelihood phylogeny for OsSAE1 (**a**) and OsSAE2 (**b**). Only nodes with bootstrap support > 75% show the correspondent bootstrap value. Os – *Oryza sativa*, Sc - *Saccharomyces cerevisiae*, At – *Arabidopsis thaliana*, Zm – *Zea mays* and Hv – *Hordeum vulgare*. **c** Venn diagram showing common CREs in the promoter region of both E1 subunit genes. Schematic representation of SAE alternative splicing forms showing three ASF for both *OsSAE1* (**d**) and *OsSAE2* (**e**). White boxes – untranslated regions (UTR); black boxes – exons; lines – introns. The proteins lengths are indicated. The difference in the sequence between *OsSAE2.1* and *OsSAE2.2/3* is indicated by an arrow. The arginine in OsSAE1 and the catalytic cysteines in OsSAE2 are depicted, as well as the domains and protein length for all ASFs. SAE2 proteins have four domains: adenylation domain where the catalytic cysteine domain is located, followed by the ubiquitin-like (UBL) and the C-terminal domains [108]. **f** Alignment of SAE2 proteins from different organisms *Saccharomyces cerevisiae*, *Arabidopsis thaliana*, and the two rice SAE2 ASF. The five amino acid insertion in OsSAE2.1 is highlighted. **g** Basal expression levels of the different ASF of *OsSAE* genes by qPCR in shoots (no pattern) and roots (patterned) in LC-93-4 (LC) and Nipponbare (Nipp). **h** and (**i**) Transcript level profile of ASFs of genes *OsSAE1* and *OsSAE2*, respectively, in response to 15 μM ABA or 100 μM of GA

**Fig. 3:**
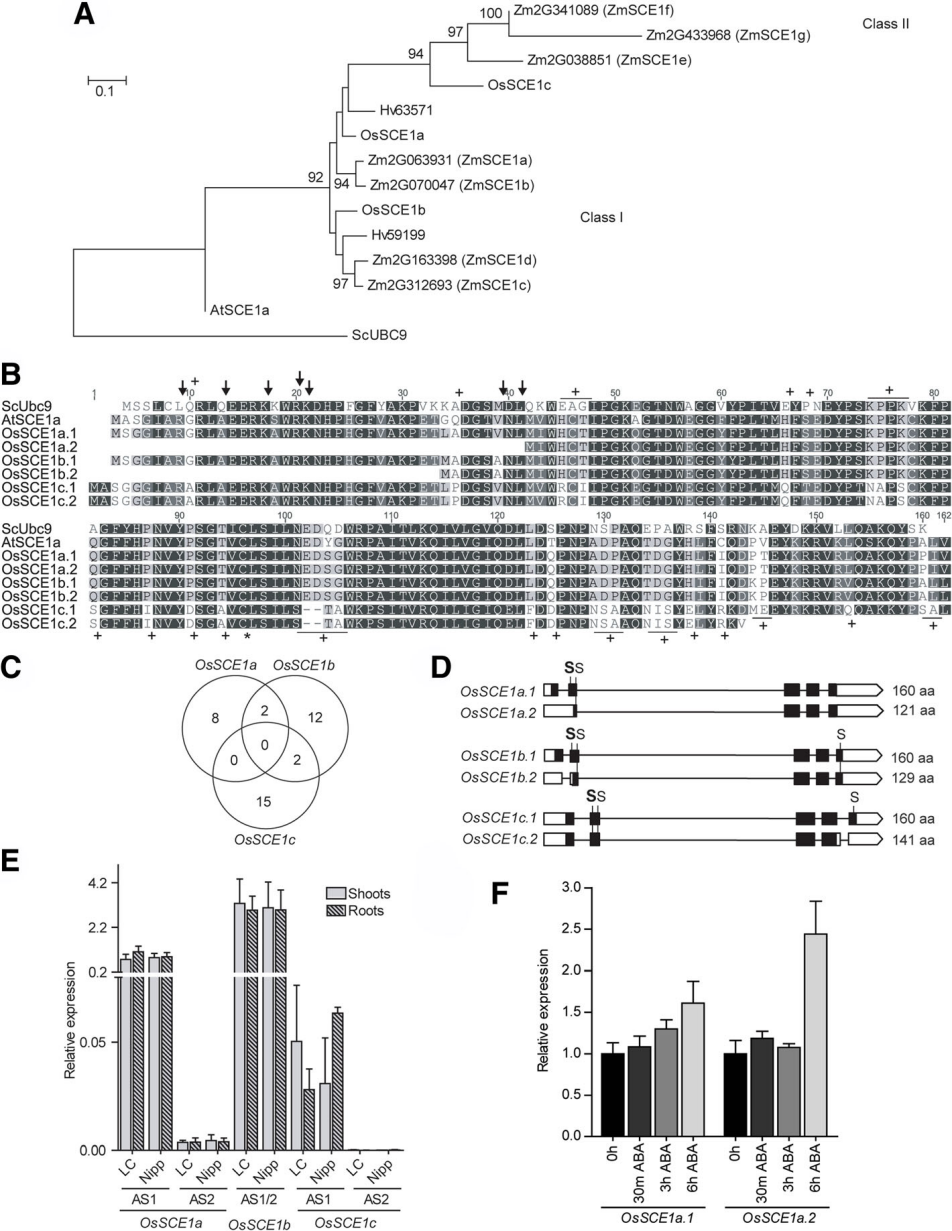
Analysis of the rice three genes of the E2 SUMO conjugating enzyme, *OsSCE1a/b/c*. **a** Maximum likelihood phylogeny of the SCE family. Only nodes with bootstrap support > 75% show the correspondent bootstrap value. Os – *Oryza sativa*, Sc - *Saccharomyces cerevisiae*, At – *Arabidopsis thaliana*, Zm – *Zea mays* and Hv – *Hordeum vulgare*. **b** Protein alignment of OsSCE1a, OsSCE1b and OsSCE1c. The catalytic cysteine in the active center is highlighted with “*”, and the E1 contact residues are highlighted with arrows. The residues that differ in Class II SCE from Class I are highlighted with “+”. **c** Venn diagram showing common CREs between OsSCEs. **d** Schematic representation of *OsSCE* alternative splicing forms, showing two for each *OsSCE*. White boxes – untranslated regions (UTR); black boxes – exons; lines – introns. The proteins lengths are indicated. High probability SUMOylation residues are indicated with an “S” in bold, whereas low probability SUMOylation residues are indicated with a normal “S”. **e** Basal expression levels of the different ASF of *OsSCE1a/b/c* genes by qPCR in shoots (no pattern) and roots (patterned) in LC-93-4 (LC) and Nipponbare (Nipp). *OsSCE1b.1* and *OsSCE1b.2* were quantified together since they could not be discriminated. **f** Transcript level profile of ASFs of *OsSCE1a* in response to 15 μM ABA

**Fig. 4:**
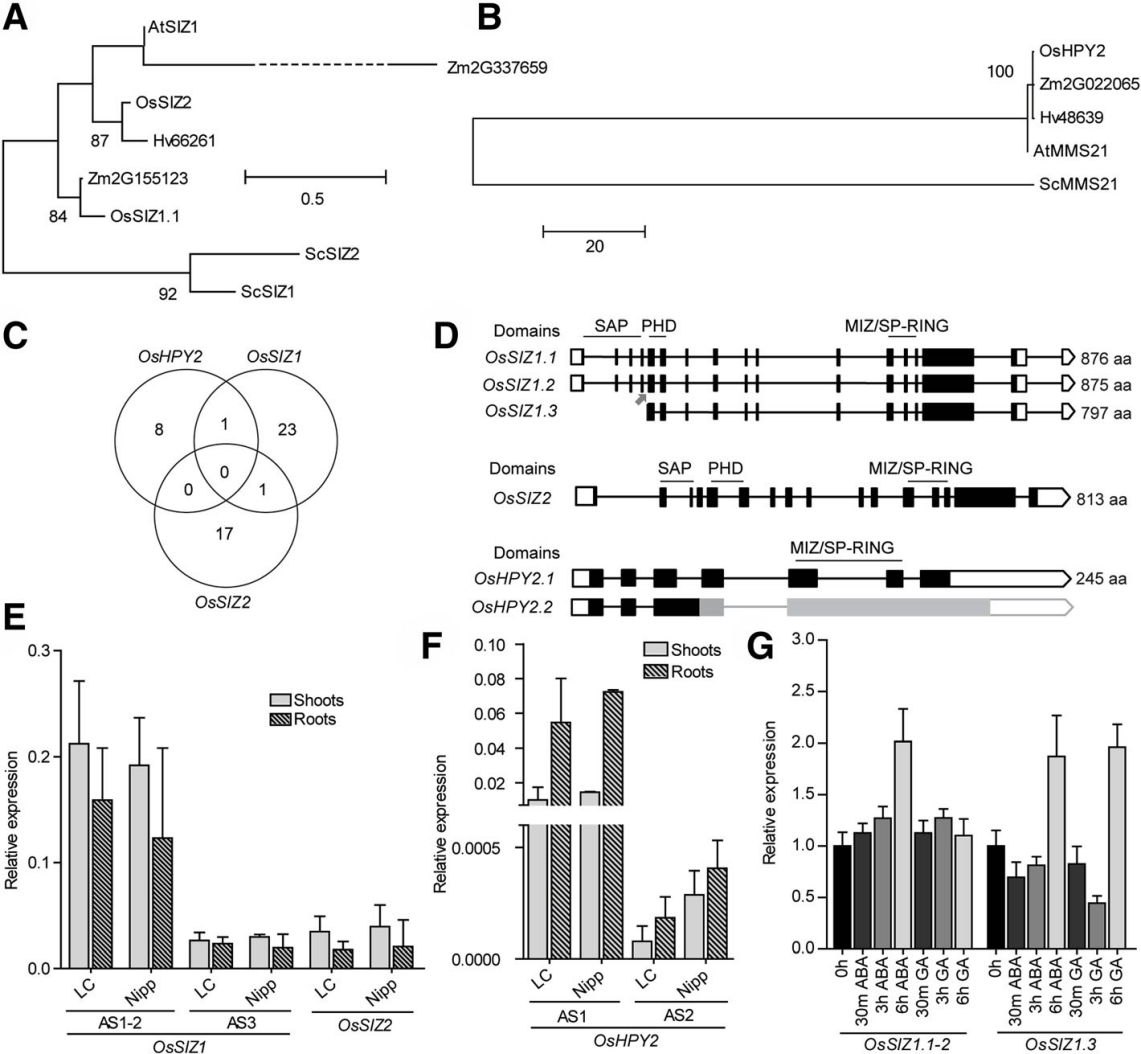
Analysis of the rice E3 SIZ and HPY2 classes. Maximum likelihood phylogeny of the E3 SUMO ligase family performed with the catalytic SP-RING domain: **a** OsSIZ and (**b**) OsHPY2. Only nodes with bootstrap support > 75% show the correspondent bootstrap value. Os – *Oryza sativa*, Sc - *Saccharomyces cerevisiae*, At – *Arabidopsis thaliana*, Zm – *Zea mays* and Hv – *Hordeum vulgare*. **c** Venn diagram showing common CREs of E3 SUMO ligases. **d** Schematic representation of E3 SUMO ligases alternative splicing forms, showing three ASF for *OsSIZ1* and two for *OsHPY2*. The difference between *OsSIZ1.1–2* is three nucleotides missing in *OsSIZ1.2* (arrow). White boxes – untranslated regions (UTR); black boxes – exons; lines – introns. The different domains and proteins lengths are indicated. In the case of *OsHPY2*, black indicates a confirmed ASF structure, which is not available for *OsHPY2.2* (in gray). Basal expression levels of *OsSIZ* (**e**) and *OsHPY2* (**f**) by qPCR in shoots (no pattern) and roots (patterned) of LC-93-4 (LC) and Nipponbare (Nipp). *OsSIZ1.1* and *OsSIZ1.2* were analysed together. **g** Transcript level profile of ASFs of *OsSIZ1* in response to 15 μM ABA or 100 μM of GA

**Fig. 5:**
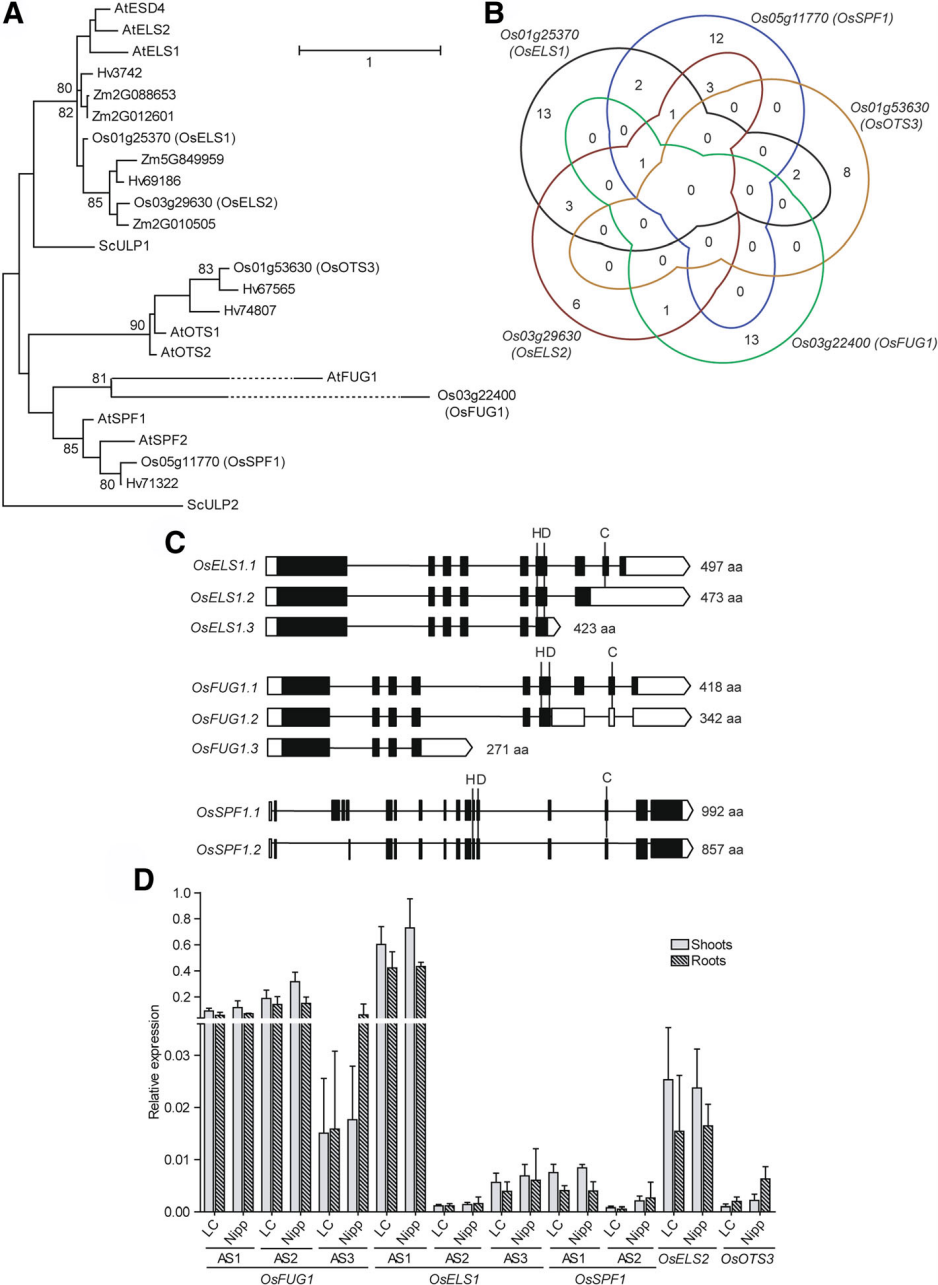
The analysed SUMO proteases in rice: Os03g22440 (OsFUG1), Os01g25370 (OsELS1), Os05g11770 (OsSPF1), Os03g29630 (OsELS2) and Os01g53630 (OsOTS3). **a** Maximum likelihood phylogeny of the SUMO proteases family. Only nodes with bootstrap support > 75% show the correspondent bootstrap value. Os – *Oryza sativa*, Sc - *Saccharomyces cerevisiae*, At – *Arabidopsis thaliana*, Zm – *Zea mays* and Hv – *Hordeum vulgare*. Only the C48 peptidase domain was used to perform the alignments. **b** Venn diagram showing common CREs between five rice SUMO proteases: Os03g22440 (OsFUG1), Os01g25370 (OsELS1), Os05g11770 (OsSPF1), Os03g29630 (OsELS2) and Os01g53630 (OsOTS3). **c** Schematic representation of the SUMO proteases alternative splicing forms, showing three alternative splicing forms for *OsFUG1* and *OsELS1*, and two for *OsSPF1*. White boxes – untranslated regions (UTR); black boxes – exons; lines – introns. Proteins lengths are indicated as well as the location of the catalytic triad of the C48 peptidase domain (histidine, aspartate and cysteine). **d** Basal expression levels of the different ASF of SUMO proteases genes performed by qPCR, in shoots (no pattern) and roots (patterned) in LC-93-4 (LC) and Nipponbare (Nipp)

In general and as expected, rice genes showed closer proximity to the genes of the two other cereals (maize and barley) in our phylogenetic analyses (Figs. 1, 2, 3, 4 and 5). In the case of the three rice SUMOs, OsSUMO1, OsSUMO2, and OsSUMO3, the phylogenetic analysis revealed that OsSUMO3 was the most divergent amongst SUMO proteins and was closely related to the least studied Arabidopsis AtSUMO5 (Fig. 1a). Indeed, OsSUMO3 showed only 46% identity with the other OsSUMO proteins (Fig. 1b). However, unlike AtSUMO5 (or AtSUMO3), OsSUMO3 has two plausible SUMOylation motifs with high/low probability (Fig. 1b), meaning that it may be possible for OsSUMO3 to form polySUMO chains. Although OsSUMO1 and OsSUMO2 are highly similar, no CREs were shared between *OsSUMO* gene promoters (Fig. 1c), a trend observed amongst all the studied rice SUMOylation machinery promoters.

Transcript level analyses revealed no differences between tissues and genotypes for *OsSUMO1* and *OsSUMO2*, indistinguishable between them, but *OsSUMO3* transcripts exist in low levels in Nipponbare (spp. *japonica*) and are almost absent in LC-93-4 (spp. *indica*) genotype. To our knowledge, *OsSUMO3* transcripts were experimentally detected for the first time, and we observed the only case of both tissue- and genotype-dependent transcript levels in our studies. In Nipponbare, *OsSUMO3* showed higher expression in roots than in shoots, while in LC-93-4, its expression in shoots was not detected under the conditions used in this study (Fig. 1d). In Arabidopsis, non-overlapping expression patterns amongst different tissues have also been reported for the four *AtSUMO* transcripts [58, 59].

#### The two rice E1 genes

The rice E1 protein complex is composed of OsSAE1 and OsSAE2 (Fig. 2a, b). Promoter region analysis revealed that both SAE gene promoters show the highest number of CREs amongst the (de)SUMOylation family (Table 1). This suggests high transcriptional regulation of both genes. Here, we found a noticeable amount of CREs related to seed-related stages and specifically to the α-amylase pathway (Additional file 1: Table S2), mainly in the *OsSAE1* promoter. Indeed, a role for SUMOylation in seed development has been extensively proven [1, 60], and so has its relationship with sugar metabolism [20]. *OsSAE2* promoter was the one with the highest amount of CREs, which are mainly involved in light regulation and abscisic acid (ABA) response (Additional file 1: Table S2), but also in seed dormancy, germination, and water stress response [61].

**Table 1:** *cis*-Acting regulatory elements (CREs) in the promoter of genes involved in the SUMOylation pathway

Protein activity	Genes	Number of different CREs present	Total number of identified CREs
SUMO	*OsSUMO1*	27	46
	*OsSUMO2*	19	27
	*OsSUMO3*	9	14
E1 SUMO activating enzyme	*OsSAE1*	25	51
	*OsSAE2*	28	92
E2 SUMO conjugation enzyme	*OsSCE1a*	10	29
	*OsSCE1b*	16	38
	*OsSCE1c*	17	22
E3 SUMO ligase	*OsHPY2*	9	17
	*OsSIZ1*	25	53
	*OsSIZ2*	18	44
SUMO protease	*OsELS1*	22	73
	*OsSPF1*	19	37
	*OsOTS3*	10	21
	*OsFUG1*	15	28
	*OsELS2*	15	21

As for putative post-transcriptional regulation, *OsSAE1* and *OsSAE2* have three alternative splicing forms each (Fig. 2d, e). ASFs found for the genes involved in SUMOylation usually differ at the 5′ or 3′ ends of their transcripts. In the case of OsSAE2, there is a five amino acid insertion at the beginning of the second exon in OsSAE2.1 that is missing in OsSAE2.2/3, but also missing in yeast and Arabidopsis (Fig. 2e, f). These results suggest that OsSAE2.2 might be the leading ASF in rice. Analysis of the basal levels of *OsSAE1* and *OsSAE2* transcripts revealed that both Nipponbare and LC-93-4 genotypes share the same levels of each transcript, as well as different tissues (shoots and roots). The similarity in transcript levels between the two subspecies is a trend in most of the analyzed genes and ASFs of the rice SUMOylation machinery. *OsSAE1* transcripts are more abundant than *OsSAE2* transcripts, with *OsSAE1.1* being the most expressed (Fig. 2g). In Arabidopsis, the transcript-profile amongst E1-related genes is similar [1].

To investigate if the presence of CREs may condition the transcriptional regulation of the E1, we subjected rice seedlings to ABA and gibberellic acid (GA) treatments. ABA and GA were chosen due to the presence of 30 ABA-related CREs in *OsSAE2*, and four GA- and one ABA-related CREs in *OsSAE1* (Additional file 1: Table S2). Indeed, when analyzing transcript behavior, the strongest transcript variation is due to ABA in *OsSAE2* transcript levels, which agrees with the observed enrichment in CREs (Fig. 2i). Also, *OsSAE1* was slightly upregulated under both ABA and GA treatment (Fig. 2h). Interestingly, several transcription factors related to ABA signaling are also SUMOylation targets [29, 30]. This may represent a regulatory feedback mechanism.

Since E1 is usually a single enzyme [51] and it is responsible for the SUMO activation step, which requires energy, one may expect that this should be the step of choice for the regulation of the global SUMOylation process. Curiously, only one CRE is shared between the genes coding for the rice E1 subunits (Fig. 2c). Literature suggests that the E1 activity may regulate global SUMOylation levels in other organisms, although the regulation at the transcript level has not been fully explored. It was recently described that E1-E2 interactions affected by mutations in AtSAE2 led to decreased SUMOylation levels and enhanced plant susceptibility to necrotrophic pathogens [62]. Jasmonic acid and ethylene are two hormones that are important in this process [63]. Interestingly, although all E2 rice genes have ethylene CREs in their promoters, only OsSCE1c has one JA-related element (Additional file 1: Table S1 and S2), which may reinforce a putative role for OsSCE1c in necrotrophic pathogen response. It is worth mentioning that Castaño-Miquel and coworkers also showed that the loss of E1-E2 interactions leads to drought susceptibility [62]. This is in line with the high amount of ABA-related CREs found in *OsSAE2* promoter from the “ABRE” family, which is one of the major families of CREs in water stress response (Additional file 1: Table S1), and is supported by the upregulation of *OsSAE2* transcripts in response to ABA treatment (Fig. 2). Also in Arabidopsis, *Atsae1* T-DNA insertion lines displayed heat and drought SUMO conjugation defects [64], suggesting that this subunit also conditions SUMOylation levels. Overall, the number and diversity of CREs found for E1 subunit genes may indicate regulation of the transcript levels of E1 genes. One might expect that SAE1 levels are kept high enough not to condition overall SUMOylation. But our data shows that *OsSAE2* transcript levels are much lower than those of *OsSAE1*, and that the promoter of *OsSAE2* does not have the diversity of CREs found in *OsSAE1* (Fig. 2). Furthermore, the transcript levels of these genes are affected by ABA and GA treatments, with *OsSAE2* being strongly upregulated with ABA (Fig. 2i). So, at this point, we believe that transcript regulation of E1 genes cannot be excluded, particularly for *OsSAE2*, but further studies need to be done to show if this upregulation results in increased levels of active protein.

#### The rice E2 family

Next, we analyzed the E2 rice gene family (*OsSCEs*). In our phylogenetic analysis, the three rice OsSCEs localized in different clusters (Fig. 3a). SCEs were recently subdivided into class I and class II type enzymes [60]. Classes were defined by the protein surface charges (with class II surface being more negative than class I); the existence of a deletion around amino acid residues 100 in class II; and some amino acid changes highlighted in Fig. 3b. The authors suggested that class II enzymes further extend the formation of SUMO chains and that the catalytic differences between classes may be a consequence of the amino acid residue changes around the active site. According to the phylogenetic analysis in Fig. 3a, rice OsSCE1a and OsSCE1b belong to class I type SCEs, while OsSCE1c belongs to class II. Indeed, OsSCE1a and OsSCE1b showed higher conservation with 93% identity at the primary sequence level, whereas OsSCE1c has 68% identity to OsSCE1a/b (Fig. 3b). The search for the presence of CREs showed almost no repeated elements amongst this family (Fig. 3c), with *OsSCE1b* showing the highest number of CREs (Table 1). Indeed, the transcript levels of *OsSCE1a*, *OsSCE1b*, and *OsSCE1c* are differentially affected by several abiotic stress conditions and throughout development. As an example, *OsSCE1b* is the only transcript showing high abundance in milky seed tissue, within the *OsSCE* genes [41, 42]. Specifically, *OsSCE1b* promoter showed CREs mainly related to light regulation and mRNA stability/transcription (Additional file 1: Table S2), which is in agreement with some known functions and targets of SUMOylation [3, 10, 32, 65].

We confirmed the existence of two putative ASF for each E2 gene. *OsSCE1a.1/2* and *OsSCE1b.1/2* show differences at the 5’-UTR, while the differences between *OsSCE1c.1* and *OsSCE1c.2* are located at the 3’-UTR (Fig. 3d). Most importantly, the loss of an N-terminal portion on the shorter proteoforms of OsSCE1a and OsSCE1b results in the removal of the residues responsible for the interaction with E1, and thus the shorter proteoforms may be inactive SUMO conjugases (Fig. 3b). Moreover, AS also affects the existence of potential SUMOylation motifs in all SCE putative proteins, which might be involved in the regulation of their activity (Fig. 3d). Transcript level analysis shows that under controlled growth conditions *OsSCE1b* exhibited the highest expression levels amongst OsSCE-genes. *OsSCE1c* transcripts were the less abundant (Fig. 3e). We tested *OsSCE1a* transcript behavior in response to ABA due to the presence of seven ABA-related CREs (Additional file 1: Table S2). We found the gene to be responsive, although only *OsSCE1a.2* showed an upregulation above the 2X threshold (Fig. 3f). So far, OsSCE1b may be the one assuring the maintenance of cellular homeostasis, OsSCE1a may be involved in ABA-related pathways, while OsSCE1c, as a class II E2, may be important for the formation of polySUMO chains.

#### Two classes of E3 genes in rice

We analyzed three rice E3 ligases: OsSIZ1, OsSIZ2, and OsHPY2. Phylogenetically, SIZ-proteins (Fig. 4a) and HPY (Fig. 4b) are unrelated. The analysis of the promoters showed only one element in common between *OsSIZ1* and *OsSIZ2*/*OsHPY2* (Fig. 4c). *OsSIZ1* promoter exhibited CREs related to light regulation and sugar metabolism (Additional file 1: Table S2), which is in agreement with the literature since AtSIZ1 has been implicated in both processes [20, 22, 32]. SUMOylation is also known to be involved in hormone signaling. Here, we detected CREs related to ABA [29], GA [31, 66], and SA signaling. The relationship between SIZ1 and SA is not new, as *AtSIZ1* knockout showed increased SA levels [23, 24, 27]. Here, we found SA-related CREs in the *OsSIZ1* promoter, but not *OsSIZ2*, suggesting that they may respond to different stimuli. Still, they may have redundant functions since their knockout/knockdown lead to similar phenotypes of defective development and reproduction [67]. Accordingly, *OsSIZ2* gene promoter showed a significant amount of seed-storage proteins from the CAATBOX1 type (Additional file 1: Table S1 and Table S2).

OsSIZ1 and OsSIZ2 both exhibit the MIZ/SP-RING domain (essential for E2 interaction), the SAP domain (thought to act as a DNA binding domain) and the PHD domain [68] (also important for SUMOylation activity [69]). *OsSIZ1* has three ASF (Fig. 4d). *OsSIZ1.1* and *OsSIZ1.2* are practically indistinguishable except for a 3 bp deletion at the 5th exon of *OsSIZ1.2* (Fig. 4d). *OsSIZ1.1*/*2* showed the highest transcript levels (Fig. 4e) and the highest CREs amount (Table 1). In line with our promoter analysis of *OsSIZ1*, which showed ABA and GA-related CREs, *OsSIZ1* was induced by both hormones (Fig. 4g). *OsSIZ1.3* behavior, 6 h after treatment, is a case where post-transcriptional regulation by alternative splicing may favor the upregulation of specific ASFs.

*OsHPY2* has two ASFs, but the sequences annotated in the rice genome databases lack the MIZ/SP-RING domain and thus, both splicing forms are incomplete. Hence, our schematic representation of *OsHPY2.2* may be inaccurate (Fig. 4d), but in our study both ASFs were detected and showed higher expression in roots as compared with shoots (Fig. 4f) in agreement with the known functions regarding the maintenance of the root stem cell niche [14].

#### The rice SUMO proteases

The rice SUMO protease family is a large one [70], and the phylogenetic analysis (Fig. 5a) helped to predict the functionality of our selected proteases [71, 72]: OsELS1(Os01g25370); OsELS2 (Os03g29630); OsSPF1 (Os05g11770); OsOTS3 (Os01g53630); and the most recently evolved OsFUG1 (Os03g22400).

Promoter analysis showed that most CREs are unique for each protease promoter (Fig. 5b), which supports their suggested functional specificity [70]. Some of the rice SUMO proteases also exhibit AS. *OsELS1* and *OsFUG1* each have three ASFs, while *OsSPF1* shows only two (Fig. 5c). However, the catalytic triad composed by a histidine, an aspartic acid, and a cysteine is only maintained in both the ASF of OsSPF1 (Additional file 1: Figure S1). In the case of OsFUG1 and OsELS1, only the longest ASFs keep the three residues and thus, might be the ones with peptidase activity. Transcript level analysis showed that *OsELS1* followed by *OsFUG1* were the most expressed under control growth conditions (Fig. 5d). *OsELS1* promoter is also the one most enriched in CREs within the family (Table 1). Found CREs are mainly involved in GA, light regulation, abiotic stress response, and seed-storage protein (Additional file 1: Table S2). We tested the effect of GA treatment on the transcription of *OsELS1* and *OsFUG1.* Overall, the transcript variations were all below a 2X threshold, except for the downregulation of the shorter ASFs of *OsELS1*. This may indicate regulation at the post-transcriptional level. We also observed a slight upregulation of all *OsFUG1* ASFs (Additional file 1: Figure S2A). The *OsOTS3* gene promoter contains light regulation-related CREs. In Arabidopsis, the double *ots1/ots2* mutants have a phenotype of hyposensitivity to red light, and AtOTS1 is capable of deSUMOylating phytochrome-B [10]. Additionally, *OsSPF1* shows an enriched promoter region in seed-storage proteins/embryo/endosperm-related CREs, supporting the phenotype obtained by the double *spf1/spf2* mutants of reduced seed fertility and altered seed size [73].

When assessing possible subcellular localization of the rice SUMOylation machinery, we found that the E1, the SUMO proteases families, and the SIZ E3 class all have nuclear localization signals (Additional file 1: Table S3). This is in agreement with the literature since these proteins were found in the nucleus [6, 37, 48, 62, 74]. However, the reason why SUMO, E2 families, and the HPY2 E3 class were the only ones lacking NLSs remains unexplained. One hypothesis may be that the α/β-importin pathway, used by the algorithm in this study, is not the mechanism by which these proteins are translocated to the nucleus [75]. The other is that it can be part of a regulatory mechanism used by the cell to further control protein (de)SUMOylation. Still, these families have been found in both cytoplasm and nucleus [7, 59, 62]. Alternative splicing did not seem to be influencing subcellular localization.

### Characterization of selected T-DNA insertion lines of the SUMOylation machinery elements

#### Molecular characterization

**Fig. 6:**
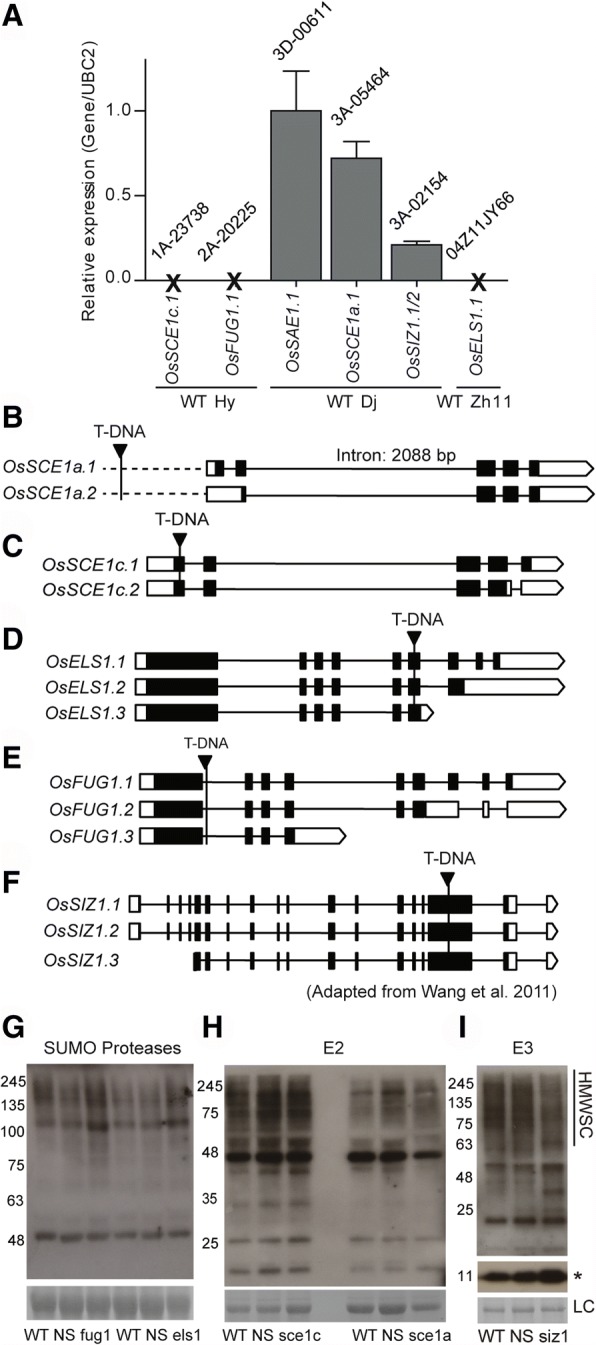
Molecular characterization of the rice T-DNA lines. **a** Expression levels of the gene of interest relative to its background line by qPCR: *OsSCE1c.1* expression in 1A-23738 vs wild type Hwayoung (Hway), *OsFUG1.1* expression in 2A-20225 vs Hwayoung; *OsSAE1.1* expression in 3D-00611 vs wild type Dongjin; *OsSCE1a.1* expression in 3A-05464 vs Dongjin; *OsSIZ1.1–2* expression in 3A-02154 vs Dongjin; *OsELS1.1* expression in 04Z11JY66 vs wild type Zhonghua11 (Zh11). Expression levels of each transcript in the T-DNA lines were normalized relative to its respective wild type (with a normalized value of 1). **b**-**f** T-DNA insertion localization site in the respective genes. White boxes – untranslated regions (UTR); black boxes – exons; lines – introns. **b** T-DNA in *OsSCE1a* knockdown (KD) line. **c** T-DNA in *OsSCE1c* knockout (KO) line. **d** T-DNA in Os*ELS1* KO line. **e** T-DNA in *OsFUG1* KO line. **f** T-DNA in *OsSIZ1* KD line is located in the 15th exon according to Wang et al. (2011). **g**-**i** Global levels of SUMOylation in the shoots of the T-DNA insertion lines in Western blots with anti-SUMO1, in normal growth conditions. T-DNA lines were compared to both wild type (WT) and negative segregant (NS) plants. Global SUMOylation levels in the KO lines of *OsFUG1* and *OsELS1* (**g**), in the T-DNA lines of *OsSCE1c* and *OsSCE1a* (**h**) and in *OsSIZ1* KD line (**i**). HMWSC – High Molecular Weight SUMO Conjugates. LC – Loading Control (Coomassie blue staining). Free SUMO is marked with an asterisk

To identify promising new tools to study the rice SUMOylation machinery, six rice T-DNA insertion lines were obtained (Table 2). Seeds were propagated, and the homozygous lines were selected by genotyping for further studies. We started by determining transcript levels of the genes affected by the T-DNA insertion and compared to the respective wild type (WT) (Fig. 6a). In the lines with T-DNA insertions in genes *OsSCE1c*, *OsFUG1*, and *OsELS1*, no transcript was detected, and thus they were considered knockout (KO) lines. For the homozygous lines of genes *OsSCE1a* and *OsSIZ1*, we observed a decrease of 30 and 80% in transcript abundance, respectively, and these will be referred to as knockdown (KD) lines. The T-DNA insertion line of *OsSAE1* showed similar *OsSAE1* transcript levels to wild-type and consequently was discarded from further analyses.

**Table 2:** T-DNA insertion lines of the rice SUMOylation machinery obtained, respective wild type and origin site

Genes Locus	Gene Name	T-DNA Line	Background	Obtained from	References
Os03g03130	*OsSCE1a*	3A-05464	Dongjin	Postech	–
Os04g49130	*OsSCE1c*	1A-23738	Hwayoung	Postech	–
Os11g30410	*OsSAE1*	3D-00611	Dongjin	Postech	–
Os05g03430	*OsSIZ1*	3A-02154	Dongjin	Postech	Wang et al. 2011
Os01g25370	*OsELS1*	04Z11JY66	Zhonghua 11	RMD	–
Os03g22400	*OsFUG1*	2A-20225	Hwayoung	Postech	–

To continue the molecular characterization of the knockout and knockdown lines, we determined the exact location of the T-DNA insertion (Fig. 6b-f). For *OsSCE1a* KD line (KD-SCE1a), the T-DNA was found 802 bp upstream from the ATG start site in the promoter region. The positioning of the T-DNA serves as an explanation for the small decrease in *OsSCE1a.1* transcript levels (Fig. 6b). For *OsSCE1c* KO line (KO-SCE1c), the T-DNA insertion is in the first exon, 250 bp from the ATG (Fig. 6c). For *OsELS1* KO line (KO-ELS1), the T-DNA insertion is in the sixth exon, 3053 bp from the ATG (Fig. 6d). The T-DNA insertion site in *OsFUG1* KO line (KO-FUG1) is in an intron, 24 bp after the end of the first exon (Fig. 6e). For *OsSIZ1* KD line, the T-DNA insertion site was previously identified in the 15th exon by Wang et al. 2011 (Fig. 6f). T-DNA insertions are usually introduced at the end of chromosomes with less frequency near the centromeres but are not biased toward a particular class of genes. However, there is a preference for the insertion site in the gene which is within the first 250 bp from the putative ATG start codon [76, 77]. Curiously, our KO-SCE1c line falls in this category (Fig. 6c).

To evaluate the effect of the T-DNA insertions in the global levels of SUMOylation under normal growth conditions, we performed Western blots using anti-AtSUMO1. Since only one transgenic line for each gene was obtained, both wild type and negative segregant (NS) plants were used to discard the influence of other possible T-DNA insertions in unknown locations or genomic rearrangements due to the transformation process. KO-ELS1 and KO-FUG1 lines showed higher SUMOylation levels than both WT and NS plants (Fig. 6g). This is in line with the data previously presented showing *OsELS1* and *OsFUG1* as the two most expressed SUMO proteases amongst the studied ones in rice (Fig. 5d). The KO*-*SCE1c line showed no differences in the global SUMOylation levels from WT and NS (Fig. 6h). The same is observed for KD-SCE1a line, although the small decrease in transcript levels could explain the absence of differences (Fig. 6h). Finally, *OsSIZ1* KD line showed lower levels of SUMOylation than WT and NS (Fig. 6i), reinforcing OsSIZ1 crucial role in the SUMOylation process. In general, the knockout of SUMO proteases leads to increased SUMOylation levels [34, 37, 78], while the knockout of *SIZ1* shows reduced levels [6, 79]. However, no variation was observed when the levels of *OsSCE1a* or *OsSCE1c* are altered (Fig. 6). Interestingly, the knockdown of the essential *AtSCE* also does not seem to significantly affect the SUMOylation levels, suggesting that minimal amounts of the E2 are sufficient, under normal growth conditions [1, 80, 81].

#### Phenotypical characterization

**Fig. 7:**
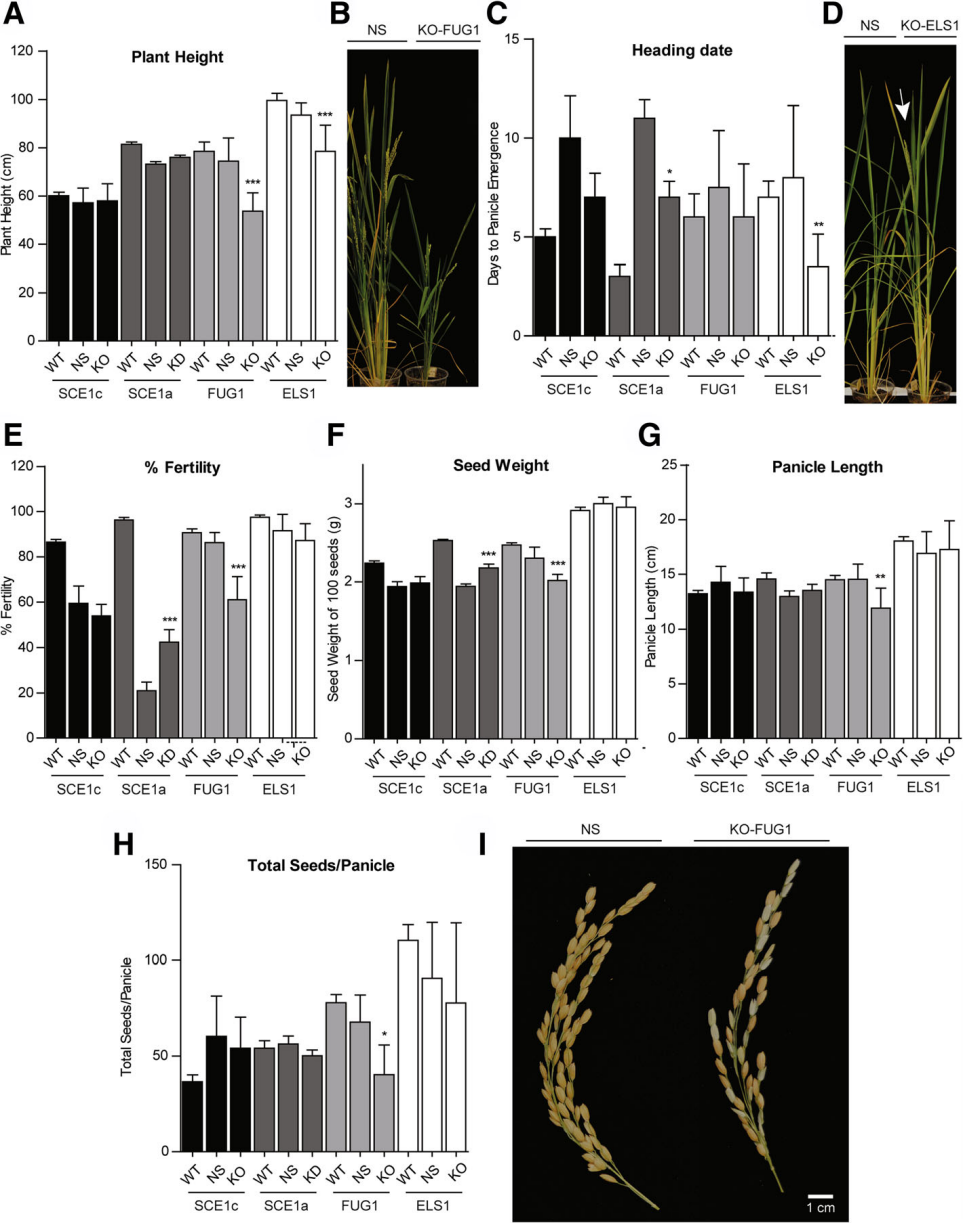
Phenotype characterization of the rice T-DNA insertion lines vs. wild type and negative segregant plants. **a** Plant height measured at reproductive stage. **b** Phenotype of *OsFUG1* knockout (KO) line and the respective negative segregant plants (NS). **c** Heading date measured as panicle ripening. The phenotype difference is exemplified by *OsELS1* KO line vs NS in (**d**). Seed-related parameters are shown in (**e**-**i**). **e** Percentage of fertility. **f** Seed weight shown as the weight of 100 seeds. **g** Panicle length (cm). **h** Total seeds per panicle. **i** Panicle phenotype of *OsFUG1* KO line versus NS. Asterisks represent statistical significance (*p-value* < 0.05). Only the significant differences between the T-DNA lines and their respective wild type/negative segregant lines are depicted

The phenotype of the *OsSIZ1* KD line was characterized elsewhere, showing development and fertility defects [82], as previously shown in Arabidopsis [53, 54]. In the phenotypic characterization, we also considered possible changes in development and seed-related parameters, since rice is a crop of major agronomical importance. Both SUMO proteases KO lines showed a significant decrease in plant height when compared to WT and NS plants (Fig. 7a), more strikingly so for KO-OsFUG1 (Fig. 7b). However, only the KO-ELS1 line showed early flowering (Fig. 7c, d). We argue that OsELS1 and AtEsd4 may be orthologs due not only to our phylogenetic analysis (Fig. 5a) but also due to their phenotype resemblance [34]. *Atesd4* plants show a pleiotropic phenotype with reduced plant size and early flowering. Also, *AtESD4* shows increased transcript levels in flowers and inflorescences under normal growth conditions [34], which also supports a function for ELS proteins in flowering stages. Still, additional KO OsELS1 lines are needed to validate these results.

The KD-SCE1a line also displayed early flowering phenotype when comparing to the NS, but late flowering when compared to the WT. The NS plants of KO-SCE1c line also showed late flowering when compared to WT (Fig. 7c). Due to the lack of accordance between their NS and WT lines, also observed for fertility rate and seed weight (Fig. 7e, f), we deemed the SCE1 T-DNA lines phenotypes inconclusive and these lines unreliable tools to study the function of the rice *SCE1* genes. This lack of consistency can be due to the presence of additional T-DNA insertions or possible genomic rearrangements. Although no hints of possible second T-DNA insertions have been detected in NS plants, it has been described that 65% of the transgenic population in question contains more than one T-DNA copy [83]. Additionally, An et al. (2005) has described in a co-segregation analysis that the inserts were not responsible for 5% of the phenotypes and that the phenotypes might have been caused by the tissue culture process that generated mutations not associated with the T-DNA [84]. Also, studies in Arabidopsis show that even the transgenic plants with straightforward genetic behavior exhibit an unexpectedly high frequency of chromosomal rearrangements, such as duplications and translocations [85] that could explain NS phenotypes.

Oppositely to the SCE T-DNA lines, both SUMO proteases T-DNA KO lines showed consistent WT and NS phenotypes. KO-FUG1 line was severely affected in its fertility rate (Fig. 7e), as well as in other seed-related parameters. We observed significant decreases in seed weight (Fig. 7f), panicle length (Fig. 7g), total seeds per panicle (Fig. 7h) and decreased number of branches per panicle (Additional file 1: Figure S3.A). Data suggests a role for *OsFUG1* in seed-related stages that influence panicle architecture and fertility (Fig. 7i). There was also an increase in the number of panicles per plant (Additional file 1: Figure S3.A/S3.B), probably as a compensation mechanism to improve plant fertility. Both SUMO proteases T-DNA insertion lines are potential tools for further investigation of *OsELS1* and *OsFUG1* roles. Due to the presence of GA-related CREs in *OsELS1* promoter, we asked if these proteases could be involved in GA signaling. We used wild type and negative segregant plants as controls, and subjected the T-DNA insertion lines to 100 μM GA. Although the differences are not statistically significant, both *OsELS1* and *OsFUG1* KO lines showed decreased internode elongation compared to the respective controls (Additional file 1: Figure S2B). This aligns with the low transcript variation observed for both genes, in response to GA (Additional file 1: Figure S2A).

Our work adds data that support the idea of functional specificity that is usually attributed to the SUMO proteases family [70]. The current view is that ESD4 proteins are important for development and flowering time, with the exception of AtELS1 (the only cytoplasmic member) whose KO only shows slightly reduced growth [35]; *OsFUG1* KO also significantly impacts development and fertility (Fig. 7); the SPF-family proteins regulate embryo development [78], and the knockout shows late flowering, increased seed size and fertility defects [73]; the OTS family is involved in salt response [48], drought [47], and copper sensitivity [86].

Due to our CREs analysis, we considered that OsELS1 and OsFUG1 might have other subcellular localization than the nucleus (Additional file 1: Table S3) since some of the CREs found in these promoters can also be found in plastid-related genes (Additional file 1: Table S1 and Table S2). We used in silico prediction software and found evidence for possible mitochondrial localization for OsFUG1 (Additional file 1: Table S4). Curiously, some mitochondrial proteins were recently described as SUMOylation targets in yeast [87] and *C. elegans* [88], and, in mammals, the SUMO E3 ligase MAPL is anchored to the mitochondria [89]. *OsFUG1* protease, which is the one that most recently evolved, showed one of the highest transcript levels (Fig. 5) and the T-DNA knockout line showed a dwarf phenotype together with severe developmental defects at the reproductive stage (Fig. 7). Still, more transgenic lines are needed to validate these results and OsFUG1 important role.

## Conclusions

In sum, the present work has underlined several potential layers of regulation for the SUMOylation process in rice. SUMOylation-related genes are regulated at the transcriptional level by several biological processes, as suggested by the analysis of promoter regions for the presence of CREs and by transcript analyses. The comparison of two rice subspecies shows that the transcription of these genes and their ASFs follows the same pattern (except for *OsSUMO3*) which suggests a strict regulation of these genes. We also provided experimental evidence for the existence of ASFs for the analyzed genes, at the transcript level, and highlighted putative control of the resulting proteoforms by post-translational modifications and differential subcellular localization, which may influence the functionality of the individual proteoforms that arise from a single gene.

We also characterized *OsELS1* and *OsFUG1* knockout lines, both with increased SUMOylation levels and non-overlapping phenotypes, which were revealed as promising tools to study the function of these genes. In fact, the so far unstudied OsFUG1 comes across as one of the most important rice SUMO proteases given its strong influence in development and fertility, while OsELS1 influences mostly flowering time.

Future studies will help to understand the importance of the several alternative splicing forms better and contribute to fully understand the role played by each of the rice SUMOylation machinery genes.

## Methods

### Identification of *cis*-acting regulatory elements (CREs)

The putative promoters (1 Kbp upstream from the putative transcription start site) of genes involved in SUMOylation process were obtained from RAP-DB database [90, 91]. We used PLANTCIS (http://www.microsatellite.org/cis_input.html) to search for CREs presence. This software uses the PLACE database [92] and performs a statistic analysis (Z-score) for the occurrences for each CRE to exclude random results. We used a cutoff of *p-value* < 0.05 to eliminate false positives [93]. The CREs were further categorized according to the literature, and each function/description is listed in Table 1, and Additional file 1: Table S1 and Table S2.

### Phylogenetic analysis

The phylogeny of the studied genes was obtained using four plant species (*Oryza sativa, Zea mays, Hordeum vulgare*, and *Arabidopsis thaliana*) and yeast (*Saccharomyces cerevisiae*) from Phytozome v10.2. All accession numbers used in this study are listed in Additional file 1: Table S5, including the ones found in *Triticum aestivum*, *Brachypodium distachyon*, *Setaria italica* and *Sorghum bicolor*. Yeast proteins were used as the outgroup. Protein sequence alignments were obtained using MUSCLE [94] and cleaned using Gblocks (allowing for smaller final blocks and less strict flanking positions) [95, 96]. For each cleaned protein alignment, we obtained the correspondent nucleotide alignment and determined the best-fit amino acid substitution model using ProtTest from MEGA6 [97]. Kimura 2-parameter model [98] together with a discrete approximation of the gamma distribution (K2 + Г) was the best-fit model for all protein families except for OsSAE1/2, and for the alignment of the SUMO proteases catalytic domain, which had Tamura 3-parameter [99] with a discrete approximation of the gamma distribution (T92 + Г) as best-fit model. With the best-fit models, we obtained maximum-likelihood (ML) phylogenies with 100 bootstrap replicates in MEGA6.

### Subcellular localization/domain and post-translational modification predictions

The tool cNLS Mapper [100] was used to search for predicted nuclear localization signals in all of the studied proteins. Available tools such as Predotar v1.3 [101] and TargetP v1.1 [102] were used to access possible mitochondrial/plastid localization signals. Sequences were also evaluated using InterPro v65.0 [103] and HMMER v2.17.3 [104] for protein family classification and for the prediction of domains and important sites. SUMOplot™ (http://www.abgent.com/sumoplot) was used to predict possible SUMOylation sites within the rice SUMOylation machinery proteins. Sequence alignments (for visualization) were performed using Geneious version 5.3.6. Statistical analysis was performed with GraphPad Prism v5.00 (*p-value* < 0.05).

### Plant growth for wild type and T-DNA insertion lines

Two rice genotypes (*Oryza sativa* L.) were used considering the two subspecies, Nipponbare (*japonica*) and LC-93-4 (*indica*). Genotype LC-93-4 (LC) was obtained from Dr. Karin Koehl (Max Planck Inst Mol Plant Physiol, Potsdam, Germany), who got them from Prof. Dr. Le Tran Binh (Institute of Biotechnology, Hanoi, Vietnam). Seeds were germinated in wet paper at 28 °C in the dark for two days and transferred to 12 h photoperiod. Seedlings were grown until nine days after germination (DAG) in MS medium supplemented with MES buffer, both half-strength, pH was adjusted to 5.1–5.2 [105]. Shoots and roots were collected separately, flash-frozen in liquid nitrogen and stored at − 80 °C for further analysis.

Rice lines with T-DNA insertions in the SUMOylation machinery genes were searched in the Salk Institute Genomic Analysis Laboratory database. Selected lines were obtained and their description/origin [83, 106] are presented in Table 2. Seeds were germinated as above. Three-week-old seedlings were genotyped using QuickExtract™ DNA Extraction Solution for DNA extraction and PCR reaction to determine homozygous, heterozygous and negative segregant plants. The primers were designed for the flanking regions of the T-DNA insertion sites for all lines. For genotyping of line 3A-05464, the primers used were OsSCE1a-F1 with either OsSCE1a-R1 or pLeftB-2715; for line 1A-23738 OsSCE1c-F1 was used with either OsSCE1c-R1 or pRightB; for line 3A-02154 OsSIZ1-F1 was used with either OsSIZ1-R1 or pLeftB-2715; for line 04Z11JY66 OsELS1-F1 was used with either OsELS1-R1 or LB-RMD; for line 2A-20225 OsFUG1-F1 was used with either OsFUG1-R1 or pLeftB-2715. For line 3D-00611, primers for the hygromycin resistance gene *hptII* were used to search for a possible T-DNA insertion. Primer sequences are listed in Additional file 1: Table S6. The T-DNA insertion sites were determined by sequencing the PCR fragment obtained with the primers corresponding to the T-DNA and the gene of interest. At least 15 plants of T2 homozygous lines were further propagated together with their respective negative segregants, and phenotyped by recording plant height, heading date (day of panicle emergence) and seed-related parameters: fertility rate, seed weight (calculated as the weight of 100 seeds), panicle length, number of branches per panicle, total seeds per panicle and panicles per plant. The leaf blades were collected, flash-frozen in liquid nitrogen and stored at − 80 °C till further analysis.

To make sure that undetected T-DNA insertions or possible genomic rearrangements did not influence the phenotype of the studied lines, we compared the homozygous lines to both their respective negative segregant and wild types plants. T-DNA homozygous and negative segregant (NS) lines were selected by: genotyping using T-DNA left/right border regions; the presence of the hygromycin resistance gene; and the inability to grow in hygromycin supplemented medium.

### Rice ABA and GA treatment

Wild type Hwayoung rice seeds were sowed and grown in hydroponic conditions as described previously. Ten 8-day-old plants were submitted to 15 μM of ABA or 100 μM of gibberellic acid 3 (Duchefa). Shoot samples were collected at 0 h (control conditions), 30 min, 3 and 6 h after hormone addition and flash-frozen till further analysis.

### Real-time reverse transcription-PCR (RT-qPCR) analysis

Shoot and root tissues were ground to a fine powder, and total RNA was extracted using Direct-zol RNA MiniPrep kit (Zymo Research). The first-strand cDNA was synthesized from 8 μg of total RNA using an anchored-oligo-(dT)_18_ primer from the Transcriptor High Fidelity cDNA Synthesis kit (Roche), according to manufacturer instructions. Quantitative PCR was performed using LightCycler 480 system and SYBR Green I Master mix (Roche, Basel, Switzerland) for a final volume of 20 μL. PCR running conditions were as follows: 95 °C for 5 min and 45 cycles of amplification at 95 °C for 10s, annealing at 56–60 °C for 10s and 72 °C for 10s. To calculate the relative transcript levels, the *Ubiquitin-conjugating enzyme E2* (*OsUBC2, LOC_Os02g42314*) transcript was used as internal control to normalize the expression data for each gene [107]. The CT values were calculated from three technical replicates, and the relative quantification of gene expression was calculated with kinetic PCR efficiency correction using the comparative Ct method [2(−ΔΔCt)] to determine the relative expression of expression/transcripts relative to endogenous control. Results represent the average calculated from four independent experiments. All primers used in this section are listed in Additional file 1: Table S7. Statistical analysis was performed using Tukey’s multiple comparison test (*p* < 0.05). Results are presented in Additional file 1: Table S8. For the hormone treatment, the average of three housekeeping genes (*OsUBC2*, *OseEF-1a* and *OsEP*) was used.

### Western blot analysis

Ground samples were lyophilized for water removal. From each sample, 4 mg were weighted, and mixed with 200 μL of Laemmli buffer (3x concentrated) using a micropestle. Samples were heated for 5 min at 95 °C and centrifuged twice at maximum speed (4 °C, 30 min). Supernatants were collected and sonicated for 10 min. From each protein extract, 1.5 μL aliquots were heated (95 °C, 5 min) and loaded into 12% SDS-PAGE. Proteins were transferred to PVDF membranes, blocked with 5% nonfat dry milk in TBS-T, and incubated with anti-AtSUMO1 (1:5000, Agrisera) overnight at 4 °C. After 1 h with the secondary antibody (anti-rabbit, 1:20,000), detection was performed with ECL (PerkinElmer). Membrane Coomassie Blue staining was used as loading control. Only freshly-prepared protein extracts were used for Western blots.

## Additional file

Additional file 1: **Table S1.** List of *cis-*acting elements of the rice SUMOylation machinery genes and respective description/function. **Table S2.** Distribution of *cis-*acting regulatory elements (CREs) according to their putative function in the promoters of the studied genes. **Table S3.** Presence of nuclear localization signals (NLSs) in the rice SUMOylation machinery proteins. Score values are present for both monopartide and bipartide NLSs by cNLS Mapper. **Table S4.** In silico prediction of the subcellular localization of OsELS1 and OsFUG1. **Table S5.** Gene locus ID of genes and species used in the phylogenetic analysis. Organisms: *Oryza sativa*, *Arabidopsis thaliana*, *Zea mays*, *Saccharomyces cerevisiae*, *Hordeum vulgare*, *Triticum aestivum*, *Brachypodium distachyon*, *Setaria italica*, *Sorghum bicolor* and *Homo sapiens*. **Table S6**. List of gene/transcript primers used for the genotyping the T-DNA insertion lines. **Table S7.** List of gene/transcript primers used in Real-time qPCR analysis. **Table S8.** Summary of the statistical analysis of rice SUMOylation machinery genes in shoots (Sh) and roots (Rt), in normal growth conditions**. Figure S1.** Alignment of the C-terminal region of the studied rice SUMO proteases. The catalytic triad is highlighted with an asterisk “*”. **Figure S2**. (A) *OsELS1* and *OsFUG1* and respective ASFs transcriptional behavior in response to 30 min, 3 h and 6 h of 100 μM of GA. Data was obtained from shoot samples of 8-day-old rice seedlings by qPCR. (B) Internode elongation (measured in cm) of seedlings subjected to 100 μM GA for 3 days at the 12-day-old stage. We used the T-DNA insertion lines of *OsELS1* and *OsFUG1*, respective wild types and negative segregant rice seedlings. A Bonferroni’s Multiple Comparison Test for the GA response data was performed (*p* < 0.05) and showed all data not significantly different. **Figure S3.** Phenotype of the T-DNA insertion lines. (A) Number of branches per panicle and (B) number of panicles per plant. Asterisks represent statistical significance (*p-value* < 0.05). Only the significant differences between the T-DNA lines and their respective wild type/negative segregant lines are depicted. (DOCX 2733 kb)

## Abbreviations

AS: Alternative splicing
ASF: Alternative splicing forms
CRE: *cis-*acting regulatory element
HPY2: High ploidy 2
KD: Knockdown
KO: Knockout
NLS: Nuclear localization signal
NS: Negative segregant
PTM: Post-translational modifications
SAE: SUMO activating enzyme
SCE: SUMO conjugating enzyme
SUMO: Small ubiquitin-like modifier
WT: Wild type
