# Supplementary material for: Insights into the transcriptional and post-transcriptional regulation of the rice SUMOylation machinery and into the role of two rice SUMO proteases
Source: BMC Plant Biol. 2018 Dec 12;18:349. doi: 10.1186/s12870-018-1547-3 (PMC6291987; doi:10.1186/s12870-018-1547-3)
Supplement: Supplementary file 1 — Table S1. List of cis-acting elements of the rice SUMOylation machinery genes and respective description/function. Table S2. Distribution of cis-acting regulatory elements (CREs) according to their putative function in the promoters of the studied genes. Table S3. Presence of nuclear localization signals (NLSs) in the rice SUMOylation machinery proteins. Score values are present for both monopartide and bipartide NLSs by cNLS Mapper. Table S4. In silico prediction of the subcellular localization of OsELS1 and OsFUG1. Table S5. Gene locus ID of genes and species used in the phylogenetic analysis. Organisms: Oryza sativa, Arabidopsis thaliana, Zea mays, Saccharomyces cerevisiae, Hordeum vulgare, Triticum aestivum, Brachypodium distachyon, Setaria italica, Sorghum bicolor and Homo sapiens. Table S6. List of gene/transcript primers used for the genotyping the T-DNA insertion lines. Table S7. List of gene/transcript primers used in Real-time qPCR analysis. Table S8. Summary of the statistical analysis of rice SUMOylation machinery genes in shoots (Sh) and roots (Rt), in normal growth conditions. Figure S1. Alignment of the C-terminal region of the studied rice SUMO proteases. The catalytic triad is highlighted with an asterisk “*”. Figure S2. (A) OsELS1 and OsFUG1 and respective ASFs transcriptional behavior in response to 30 min, 3 h and 6 h of 100 μM of GA. Data was obtained from shoot samples of 8-day-old rice seedlings by qPCR. (B) Internode elongation (measured in cm) of seedlings subjected to 100 μM GA for 3 days at the 12-day-old stage. We used the T-DNA insertion lines of OsELS1 and OsFUG1, respective wild types and negative segregant rice seedlings. A Bonferroni’s Multiple Comparison Test for the GA response data was performed (p < 0.05) and showed all data not significantly different. Figure S3. Phenotype of the T-DNA insertion lines. (A) Number of branches per panicle and (B) number of panicles per plant. Asterisks represent statistical significance (p-value < [file 12870_2018_1547_MOESM1_ESM.docx]

**Additional File 1**

**Supplemental Tables**

**Table S1 –** List of *cis-*acting elements of the rice SUMOylation machinery genes and respective description/function.

| ***cis-*acting elements** | **Total** | **Description of *OsSAE1* *cis-*elements** |
| --- | --- | --- |
| ABREOSRAB21 | 1 | ABA-responsive element/Rab21-related expression |
| AMYBOX1 | 1 | Sugar-responsive element (alpha-amylase-related expression) |
| AMYBOX2 | 2 | Sugar-responsive element (alpha-amylase-related expression) |
| ANAERO3CONSENSUS | 1 | Fermentative pathway |
| AUXREPSIAA4 | 1 | Auxin-responsive element |
| BOXIINTPATPB | 2 | Found in plastid genes |
| CATATGGMSAUR | 3 | Auxin-responsive element |
| CCA1ATLHCB1 | 1 | Regulation by phytochrome |
| CEREGLUBOX3PSLEGA | 1 | Storage-protein element |
| CRTDREHVCBF2 | 1 | Temperature-regulation element |
| GAREAT | 1 | GA-responsive element |
| GGTCCCATGMSAUR | 1 | Auxin-responsive gene |
| GT1GMSCAM4 | 4 | Pathogen/salt-induced expression |
| LECPLEACS2 | 1 | Ethylene biosynthesis |
| LTRE1HVBLT49 | 1 | Low temperature-responsive element |
| MYBATRD22 | 1 | Dehydration-responsive element (Rd22-related expression) |
| MYBGAHV | 1 | GA regulation/Sugar metabolism (alpha-amylase-related expression) |
| POLASIG1 | 4 | mRNA polyadenylation/Seed germination (alpha-amylase-related expression) |
| POLLEN1LELAT52 | 6 | Pollen-expression element |
| RHERPATEXPA7 | 2 | Root hair-specific cis-elements |
| SORLIP2AT | 4 | Light-responsive element |
| TATCCACHVAL21 | 2 | GA regulation |
| TATCCAOSAMY | 4 | Seed germination/Sugar metabolism (alpha-amylase-related expression) |
| TATCCAYMOTIFOSRAMY3D | 4 | Seed germination/Sugar metabolism (alpha-amylase-related expression) |
| TL1ATSAR | 1 | Pathogen-response |
| ***cis-*acting elements** | **Total** | **Description of *OsSAE2* *cis-*elements** |
| ABREATCONSENSUS | 4 | ABA-responsive element |
| ABREATRD22 | 1 | ABA-responsive element/ Rd22-related expression |
| ABRELATERD1 | 5 | ABA-responsive element/Erd1-related expression (early responsive to dehydration) |
| ABRERATCAL | 4 | Light-responsive element |
| ABREZMRAB28 | 4 | ABA and water stress responses/ABA-inducible in embryos and vegetative tissues |
| ACGTABREMOTIFA2OSEM | 4 | ABA-responsive element |
| ACGTATERD1 | 7 | ABA-responsive element/Erd1-related expression (early responsive to dehydration) |
| ACGTOSGLUB1 | 1 | Endosperm-specific element |
| ACGTROOT1 | 4 | Root-related expression |
| ANAERO2CONSENSUS | 2 | Fermentative pathway |
| BOXIIPCCHS | 4 | Light Regulation |
| CACGTGMOTIF | 4 | Light-responsive element/Required for embryogenesis |
| DRE2COREZMRAB17 | 1 | ABA-inducible /Rab17-related expression during late embryogenesis |
| EMBP1TAEM | 4 | ABA-mediated stress-signaling pathway |
| GBOXLERBCS | 4 | Light regulation |
| GCCCORE | 3 | Ethylene-responsive element/biotic stress |
| IRO2OS | 4 | Iron metabolism |
| LRENPCABE | 3 | Light Regulation |
| LTREATLTI78 | 1 | Low temperature-responsive element |
| MYBPZM | 3 | Floral organs |
| QELEMENTZMZM13 | 1 | Pollen-expression element |
| SORLIP1AT | 7 | Light-responsive element |
| SORLIP2AT | 9 | Light-responsive element |
| TATABOX1 | 1 | Seed germination/Sugar metabolism (alpha-amylase-related expression) |
| TATABOX3 | 1 | Sugar metabolism |
| UP1ATMSD | 1 | Found after main stem decapitation |
| UPRMOTIFIIAT | 3 | Unfolded protein response |
| WBOXNTCHN48 | 2 | Required for elicitor responsiveness |
| ***cis-*acting elements** | **Total** | **Description of *OsSCE1a* *cis-*elements** |
| ACGTTBOX | 1 | Root-related expression |
| CCAATBOX1 | 3 | Heat-responsive element |
| E2FCONSENSUS | 2 | Cell cycle regulation/DNA replication |
| ELRECOREPCRP1 | 1 | Elicitor-responsive proteins/biotic stress |
| GCBP2ZMGAPC4 | 2 | Anaerobic gene expression |
| LTRECOREATCOR15 | 7 | Low temperature-responsive element/ABA-responsive |
| NAPINMOTIFBN | 1 | Seed storage protein |
| PYRIMIDINEBOXOSRAMY1A | 2 | Sugar-responsive element (alpha-amylase-related expression)/GA regulation |
| SITEIIATCYTC | 4 | Anther- and meristem-specific element |
| SORLIP2AT | 6 | Light-responsive element |
| ***cis-*acting elements** | **Total** | **Description of *OsSCE1b* *cis-*elements** |
| ABRERATCAL | 2 | Light-responsive element |
| ACGTTBOX | 1 | Root-related expression |
| ARR1AT | 9 | Response regulators |
| BOXIINTPATPB | 2 | Found in plastid genes |
| INRNTPSADB | 3 | Light Regulation |
| LECPLEACS2 | 1 | Ethylene biosynthesis |
| LTRE1HVBLT49 | 1 | Low temperature-responsive element |
| POLASIG1 | 4 | mRNA polyadenylation/Seed germination (alpha-amylase-related expression) |
| POLASIG3 | 5 | mRNA polyadenylation |
| PYRIMIDINEBOXHVEPB1 | 1 | GA regulation |
| REBETALGLHCB21 | 1 | Light regulation |
| SORLIP2AT | 3 | Light-responsive element |
| SORLREP2AT | 1 | Light-responsive element |
| TATABOX3 | 1 | Sugar metabolism |
| TRANSINITDICOTS | 1 | Context sequence of translational initiation codon in dicots |
| WBBOXPCWRKY1 | 2 | Required for elicitor responsiveness/regulation of early defense-response genes |
| ***cis-*acting elements** | **Total** | **Description of *OsSCE1c* *cis-*elements** |
| AUXREPSIAA4 | 1 | Auxin-responsive element |
| BS1EGCCR | 1 | Vascular expression |
| CTRMCAMV35S | 1 | Can enhance gene expression |
| DRE2COREZMRAB17 | 1 | ABA-inducible /Rab17-related expression during late embryogenesis |
| GGTCCCATGMSAUR | 1 | Auxin-responsive gene |
| GT1CORE | 1 | Light Regulation |
| HEXMOTIFTAH3H4 | 1 | DNA binding/Found in histone proteins |
| LECPLEACS2 | 2 | Ethylene biosynthesis |
| MRNASTA1CRPSBD | 1 | mRNA stability determinant |
| MYCATERD1 | 2 | Storage-protein element; Erd1-related expression (early response to dehydration) |
| RAV1BAT | 1 | Rosette leaves and roots-specific element |
| SEF1MOTIF | 1 | Embryo-specific element |
| SP8BFIBSP8BIB | 2 | Sugar metabolism |
| SURECOREATSULTR11 | 3 | Sulfur-responsive element |
| T/GBOXATPIN2 | 1 | JA-responsive element |
| TATABOX3 | 1 | Sugar metabolism |
| UPRMOTIFIIAT | 1 | Unfolded protein response |
| ***cis-*acting elements** | **Total** | **Description of *OsSIZ1* *cis-*elements** |
| ABREATCONSENSUS | 1 | ABA-responsive element |
| ABRELATERD1 | 3 | ABA-responsive element/Erd1-related expression (early responsive to dehydration) |
| ABREOSRAB21 | 1 | ABA-responsive element/Rab21-related expression |
| ABREZMRAB28 | 1 | ABA and water stress responses/ABA-inducible in embryos and vegetative tissues |
| ACGTABOX | 1 | Sugar repression-responsive element |
| ACGTABREMOTIFA2OSEM | 3 | ABA-responsive element |
| ACGTATERD1 | 7 | ABA-responsive element/Erd1-related expression (early responsive to dehydration) |
| ASF1MOTIFCAMV | 3 | Auxin- and salicylic acid-responsive element/Light-responsive element |
| AUXRETGA2GMGH3 | 1 | Auxin-responsive element |
| BOXIIPCCHS | 3 | Light Regulation |
| ELRECOREPCRP1 | 1 | Elicitor-responsive proteins/biotic stress |
| EMBP1TAEM | 1 | ABA-mediated stress-signaling pathway |
| GBOXLERBCS | 1 | Light regulation |
| HDZIP2ATATHB2 | 1 | Light Regulation |
| HEXAT | 1 | DNA binding; bZIP protein |
| IRO2OS | 1 | Iron metabolism |
| LREBOXIIPCCHS1 | 1 | Light-responsive element |
| LRENPCABE | 2 | Light Regulation |
| SEF3MOTIFGM | 2 | Embryo-specific element |
| TBOXATGAPB | 2 | Light regulation |
| TGACGTVMAMY | 1 | Seed germination/Sugar metabolism (alpha-amylase-related expression) |
| TRANSINITDICOTS | 1 | Context sequence of translational initiation codon in dicots |
| WBOXATNPR1 | 3 | SA-induced element |
| WBOXNTERF3 | 4 | Wounding-responsive element |
| WRKY71OS | 7 | GA regulation |
| ***cis-*acting elements** | **Total** | **Description of *OsSIZ2* *cis-*elements** |
| ARFAT | 1 | Auxin-responsive gene |
| CAATBOX1 | 10 | Storage-protein element |
| CARGCW8GAT | 3 | Flowering-time |
| CARGNCAT | 1 | Flowering-time/GA metabolism |
| CCA1ATLHCB1 | 1 | Regulation by phytochrome |
| CCAATBOX1 | 3 | Heat-responsive element |
| GLMHVCHORD | 1 | Nitrogen metabolism |
| HDZIP2ATATHB2 | 1 | Light Regulation |
| L1BOXATPDF1 | 1 | Regulation of shoot epidermal cell differentiation |
| MYB2AT | 1 | Water stress-responsive |
| NODCON2GM | 4 | Nodules-expression element |
| OSE2ROOTNODULE | 4 | Root nodules-expression element |
| SP8BFIBSP8BIB | 1 | Sugar metabolism |
| TAAAGSTKST1 | 4 | Guard cells expression element |
| TATABOX2 | 2 | Seed germination/Storage-protein/Sugar metabolism |
| TATABOX3 | 1 | Sugar metabolism |
| TELOBOXATEEF1AA1 | 2 | Telomers/Root primordia |
| UP2ATMSD | 3 | Found after main stem decapitation |
| ***cis-*acting elements** | **Total** | **Description of *OsHPY2* *cis-*elements** |
| ACGTTBOX | 1 | Root-related expression |
| AGMOTIFNTMYB2 | 1 | Wounding or elicitor treatment element |
| EECCRCAH1 | 3 | Carbon-concentrating mechanism |
| ELRECOREPCRP1 | 1 | Elicitor-responsive proteins/biotic stress |
| GT1CORE | 3 | Light Regulation |
| MYCATRD22 | 3 | Storage-protein element; Rd22-related expression (dehydration-responsive element) |
| QELEMENTZMZM13 | 1 | Pollen-expression element |
| RBCSCONSENSUS | 1 | Light regulation |
| SITEIIATCYTC | 3 | Anther- and meristem-specific element |
| ***cis-*acting elements** | **Total** | **Description of *OsELS1* *cis-*elements** |
| ABREOSRAB21 | 1 | ABA-responsive element/Rab21-related expression |
| ACGTABREMOTIFA2OSEM | 1 | ABA-responsive element |
| BOXIIPCCHS | 1 | Light Regulation |
| BS1EGCCR | 1 | Vascular expression |
| CAATBOX1 | 9 | Storage-protein element |
| CARGNCAT | 1 | Flowering-time/GA metabolism |
| DRE2COREZMRAB17 | 1 | ABA-inducible /Rab17-related expression during late embryogenesis |
| EBOXBNNAPA | 10 | Storage-protein element |
| ELRECOREPCRP1 | 1 | Elicitor-responsive proteins/biotic stress |
| GT1CONSENSUS | 8 | Light-responsive element |
| HDZIP2ATATHB2 | 1 | Light Regulation |
| LRENPCABE | 1 | Light Regulation |
| MYB1LEPR | 1 | Pathogen response |
| MYCCONSENSUSAT | 10 | Rd22-related expression (dehydration-responsive element)/Cold-responsive |
| PRECONSCRHSP70A | 5 | Plastid-response element/Induction of HSPs |
| PYRIMIDINEBOXHVEPB1 | 1 | GA regulation |
| RAV1AAT | 6 | Rosette leaves and roots-specific element |
| SURECOREATSULTR11 | 3 | Sulfur-responsive element |
| T/GBOXATPIN2 | 1 | JA-responsive element |
| TRANSINITDICOTS | 1 | Context sequence of translational initiation codon in dicots |
| TRANSINITMONOCOTS | 2 | Context sequence of translational initiation codon in monocots |
| WRKY71OS | 7 | GA regulation |
| ***cis-*acting elements** | **Total** | **Description of *OsSPF1* *cis-*elements** |
| ACGTABOX | 1 | Sugar repression-responsive element |
| ACGTTBOX | 1 | Root-related expression |
| AMMORESIVDCRNIA1 | 1 | Nitrogen metabolism |
| AMYBOX1 | 1 | Sugar-responsive element (alpha-amylase-related expression) |
| CAATBOX1 | 10 | Storage-protein element |
| CARGCW8GAT | 3 | Flowering-time |
| ELRECOREPCRP1 | 1 | Elicitor-responsive proteins/biotic stress |
| GAREAT | 2 | GA-responsive element |
| GT1CORE | 1 | Light Regulation |
| INRNTPSADB | 3 | Light Regulation |
| LTRE1HVBLT49 | 1 | Low temperature-responsive element |
| MARABOX1 | 1 | Scaffold attachment sites |
| MARARS | 2 | Scaffold attachment sites |
| MARTBOX | 2 | Scaffold attachment sites |
| MYBGAHV | 1 | GA regulation/Sugar metabolism (alpha-amylase-related expression) |
| SEF1MOTIF | 1 | Embryo-specific element |
| TATABOX2 | 2 | Seed germination/Storage-protein/Sugar metabolism |
| TRANSINITDICOTS | 1 | Context sequence of translational initiation codon in dicots |
| TRANSINITMONOCOTS | 2 | Context sequence of translational initiation codon in monocots |
| ***cis-*acting elements** | **Total** | **Description of *OsOTS3* *cis-*elements** |
| ABREOSRAB21 | 1 | ABA-responsive element/Rab21-related expression |
| AMMORESIIUDCRNIA1 | 1 | Nitrogen metabolism |
| ARFAT | 1 | Auxin-responsive gene |
| ASF1MOTIFCAMV | 2 | Auxin- and salicylic acid-responsive element/Light-responsive element |
| CIACADIANLELHC | 2 | Circadian-expression element |
| DRE2COREZMRAB17 | 1 | ABA-inducible /Rab17-related expression during late embryogenesis |
| GTGANTG10 | 7 | Pollen-expression element |
| L1BOXATPDF1 | 1 | Regulation of shoot epidermal cell differentiation |
| PALBOXAPC | 2 | Elicitor or light responsiveness |
| POLASIG3 | 3 | mRNA polyadenylation |
| ***cis-*acting elements** | **Total** | **Description of *OsFUG1 cis-*elements** |
| AMYBOX2 | 1 | Sugar-responsive element (alpha-amylase-related expression) |
| CCAATBOX1 | 3 | Heat-responsive element |
| ERELEE4 | 1 | Ethylene-responsive element/senescence-related element |
| LECPLEACS2 | 2 | Ethylene biosynthesis |
| MRNASTA2CRPSBD | 2 | mRNA stability determinant |
| MYB2AT | 1 | Water stress-responsive |
| MYCATERD1 | 2 | Storage-protein element; Erd1-related expression (early response to dehydration) |
| ROOTMOTIFTAPOX1 | 6 | Higher root expression |
| S1FBOXSORPS1L21 | 4 | Plastid-ribosomal expression |
| S1FSORPL21 | 1 | Plastid-ribosomal expression |
| SP8BFIBSP8BIB | 1 | Sugar metabolism |
| SV40COREENHAN | 1 | Light Regulation |
| TATABOX3 | 1 | Sugar metabolism |
| TATCCAYMOTIFOSRAMY3D | 1 | Seed germination/Sugar metabolism (alpha-amylase-related expression) |
| TRANSINITDICOTS | 1 | Context sequence of translational initiation codon in dicots |
| ***cis-*acting elements** | **Total** | **Description of *OsELS2* *cis-*elements** |
| AACACOREOSGLUB1 | 2 | Endosperm-specific element |
| AMYBOX1 | 1 | Sugar-responsive element (alpha-amylase-related expression) |
| ELRECOREPCRP1 | 1 | Elicitor-responsive proteins/biotic stress |
| GAREAT | 1 | GA-responsive element |
| INTRONLOWER | 2 | 3' Intron-exon splice junctions |
| MYB1LEPR | 1 | Pathogen response |
| MYBGAHV | 1 | GA regulation/Sugar metabolism (alpha-amylase-related expression) |
| NAPINMOTIFBN | 1 | Seed storage protein |
| POLASIG2 | 2 | mRNA polyadenylation/Seed germination (alpha-amylase-related expression) |
| PYRIMIDINEBOXHVEPB1 | 1 | GA regulation |
| RAV1AAT | 4 | Rosette leaves and roots-specific element |
| SORLIP5AT | 1 | Light-responsive element |
| TATABOX3 | 1 | Sugar metabolism |
| TRANSINITDICOTS | 1 | Context sequence of translational initiation codon in dicots |
| WUSATAg | 1 | Maintenance of the stem cells (QC cells) |
| ***cis-*acting elements** | **Total** | **Description of *OsSUMO1* *cis-*elements** |
| AACACOREOSGLUB1 | 1 | Endosperm-specific element |
| AMYBOX1 | 1 | Sugar-responsive element (alpha-amylase-related expression) |
| ANAERO1CONSENSUS | 2 | Fermentative pathway |
| ASF1MOTIFCAMV | 3 | Auxin- and salicylic acid-responsive element/Light-responsive element |
| CGCGBOXAT | 6 | Signaling pathways |
| DPBFCOREDCDC3 | 4 | Embryo-specific element/ABA-responsive |
| E2FANTRNR | 1 | Cell cycle regulation/DNA replication |
| E2FBNTRNR | 1 | Cell cycle regulation/DNA replication/UV-C radiation |
| E2FCONSENSUS | 2 | Cell cycle regulation/DNA replication |
| ELRECOREPCRP1 | 1 | Elicitor-responsive proteins/biotic stress |
| GAREAT | 1 | GA-responsive element |
| IBOX | 1 | Light regulation |
| IBOXCORENT | 1 | Light regulation |
| MYB1AT | 3 | Dehydration-responsive element (Rd22-related expression) |
| MYB2CONSENSUSAT | 2 | Dehydration-responsive element (Rd22-related expression) |
| MYBCOREATCYCB1 | 4 | Cell cycle regulation |
| MYBGAHV | 1 | GA regulation/Sugar metabolism (alpha-amylase-related expression) |
| PROLAMINBOXOSGLUB1 | 1 | Endosperm-specific element |
| PYRIMIDINEBOXHVEPB1 | 1 | GA regulation |
| RAV1BAT | 1 | Rosette leaves and roots-specific element |
| REALPHALGLHCB21 | 2 | Light regulation |
| RHERPATEXPA7 | 2 | Root hair-specific cis-elements |
| TATAPVTRNALEU | 1 | Transcription initiation |
| TGACGTVMAMY | 1 | Seed germination/Sugar metabolism (alpha-amylase-related expression) |
| VOZATVPP | 1 | Pollen-specific element |
| ***cis-*acting elements** | **Total** | **Description of *OsSUMO2* *cis-*elements** |
| -300CORE | 1 | Endosperm-specific element |
| AMYBOX2 | 1 | Sugar-responsive element (alpha-amylase-related expression) |
| BS1EGCCR | 1 | Vascular expression |
| ELRECOREPCRP1 | 1 | Elicitor-responsive proteins/biotic stress |
| EMHVCHORD | 1 | Endosperm-specific element/nitrogen metabolism |
| GT1CORE | 1 | Light Regulation |
| IBOX | 1 | Light regulation |
| IBOXCORE | 3 | Light regulation |
| MARARS | 1 | Scaffold attachment sites |
| MYBST1 | 3 | Transcriptional activator |
| PYRIMIDINEBOXOSRAMY1A | 2 | Sugar-responsive element (alpha-amylase-related expression)/GA regulation |
| RAV1AAT | 4 | Rosette leaves and roots-specific element |
| RAV1BAT | 1 | Rosette leaves and roots-specific element |
| SEF1MOTIF | 1 | Embryo-specific element |
| TATCCAOSAMY | 2 | Seed germination/Sugar metabolism (alpha-amylase-related expression) |
| TATCCAYMOTIFOSRAMY3D | 1 | Seed germination/Sugar metabolism (alpha-amylase-related expression) |
| UPRMOTIFIIAT | 1 | Unfolded protein response |
| VOZATVPP | 1 | Pollen-specific element |
| ***cis-*acting elements** | **Total** | **Description of *OsSUMO3 cis-*elements** |
| BIHD1OS | 3 | Homeodomain transcriptional factors/possible biotic stress response |
| ERELEE4 | 1 | Ethylene-responsive element/senescence-related element |
| LTRE1HVBLT49 | 1 | Low temperature-responsive element |
| POLASIG2 | 2 | mRNA polyadenylation/Seed germination (alpha-amylase-related expression) |
| REBETALGLHCB21 | 1 | Light regulation |
| SORLIP2AT | 3 | Light-responsive element |
| TATABOXOSPAL | 2 | Transcription initiation |
| UPRMOTIFIIAT | 1 | Unfolded protein response |

**Table S2 –** Distribution of *cis-*acting regulatory elements (CREs) according to their putative function in the promoters of the studied genes.

| Elements/  CREs description | E1 | | E2 | | | E3 | | | SUMO protease | | | | | SUMO | | |  |
| --- | --- | --- | --- | --- | --- | --- | --- | --- | --- | --- | --- | --- | --- | --- | --- | --- | --- |
|  | *OsSAE1* | *OsSAE2* | *OsSCE1a* | *OsSCE1b* | *OsSCE1c* | *OsHPY2* | *OsSIZ1* | *OsSIZ2* | *OsELS1* | *OsSPF1* | *OsOTS3* | *OsFUG1* | *OsELS2* | *OsSUMO1* | *OsSUMO2* | *OsSUMO3* | Total |
| **Hormones** |  |  |  |  |  |  |  |  |  |  |  |  |  |  |  |  | 138 |
| Ethylene | 1 | 3 | 0 | 1 | 2 | 0 | 0 | 0 | 0 | 0 | 0 | 3 | 0 | 0 | 0 | 1 | 11 |
| Auxin | 5 | 0 | 0 | 0 | 2 | 0 | 4 | 1 | 0 | 0 | 3 | 0 | 0 | 3 | 0 | 0 | 18 |
| Salicylic acid | 0 | 0 | 0 | 0 | 0 | 0 | 6 | 0 | 0 | 0 | 2 | 0 | 0 | 3 | 0 | 0 | 11 |
| Gibberillic acid | 4 | 0 | 0 | 1 | 0 | 0 | 7 | 1 | 8 | 3 | 0 | 0 | 2 | 3 | 2 | 0 | 31 |
| Abscisic acid | 1 | 30 | 7 | 0 | 1 | 0 | 17 | 0 | 3 | 0 | 2 | 0 | 0 | 4 | 0 | 0 | 65 |
| Jasmonic acid | 0 | 0 | 0 | 0 | 1 | 0 | 0 | 0 | 1 | 0 | 0 | 0 | 0 | 0 | 0 | 0 | 2 |
| **Environmental Stress** |  |  |  |  |  |  |  |  |  |  |  |  |  |  |  |  | 120 |
| Water stress | 1 | 0 | 0 | 0 | 2 | 3 | 0 | 1 | 10 | 0 | 0 | 3 | 0 | 5 | 0 | 0 | 25 |
| ABA/Water stress | 1 | 18 | 0 | 0 | 1 | 0 | 12 | 0 | 2 | 0 | 2 | 0 | 0 | 0 | 0 | 0 | 36 |
| Heat | 0 | 0 | 3 | 0 | 0 | 0 | 0 | 3 | 5 | 0 | 0 | 3 | 0 | 0 | 0 | 0 | 14 |
| Cold | 1 | 1 | 7 | 1 | 0 | 0 | 0 | 0 | 10 | 1 | 0 | 0 | 0 | 0 | 0 | 1 | 22 |
| Biotic stress | 5 | 3 | 1 | 2 | 0 | 1 | 1 | 0 | 2 | 1 | 0 | 0 | 2 | 1 | 1 | 3 | 23 |
| **Light Regulation** | 5 | 35 | 6 | 10 | 1 | 4 | 13 | 2 | 11 | 4 | 4 | 1 | 1 | 6 | 5 | 4 | 112 |
| **Nutrient Metabolism** | 17 | 8 | 4 | 5 | 6 | 3 | 3 | 5 | 3 | 6 | 1 | 4 | 5 | 6 | 7 | 0 | 83 |
| Sugars | 16 | 2 | 2 | 5 | 3 | 0 | 2 | 5 | 0 | 5 | 0 | 4 | 5 | 3 | 6 | 0 | 58 |
| **Organ-specific** |  |  |  |  |  |  |  |  |  |  |  |  |  |  |  |  | 235 |
| Roots | 2 | 4 | 1 | 1 | 0 | 1 | 0 | 10 | 0 | 1 | 0 | 6 | 0 | 2 | 0 | 0 | 28 |
| Shoots | 0 | 0 | 0 | 0 | 0 | 0 | 0 | 8 | 0 | 0 | 1 | 0 | 0 | 0 | 0 | 0 | 9 |
| Shoots and Roots | 0 | 0 | 0 | 0 | 1 | 0 | 0 | 0 | 6 | 0 | 0 | 0 | 4 | 1 | 5 | 0 | 17 |
| Vascular | 0 | 0 | 0 | 0 | 1 | 0 | 0 | 0 | 1 | 0 | 0 | 0 | 0 | 0 | 1 | 0 | 3 |
| **Seed-related Stages** |  |  |  |  |  |  |  |  |  |  |  |  |  |  |  |  | 178 |
| Seed-storage proteins/ Embryo/Endosperm | 13 | 7 | 1 | 4 | 4 | 3 | 3 | 12 | 19 | 13 | 0 | 3 | 5 | 7 | 6 | 0 | 100 |
| alfa-amylase | 16 | 1 | 2 | 4 | 0 | 0 | 1 | 0 | 0 | 2 | 0 | 2 | 3 | 3 | 6 | 2 | 42 |
| Pollen | 6 | 1 | 0 | 0 | 0 | 3 | 0 | 0 | 0 | 0 | 7 | 0 | 0 | 1 | 1 | 0 | 19 |
| Flowering | 0 | 3 | 4 | 0 | 0 | 3 | 0 | 4 | 0 | 3 | 0 | 0 | 0 | 0 | 0 | 0 | 17 |
| **Cell-specific** |  |  |  |  |  |  |  |  |  |  |  |  |  |  |  |  | 67 |
| mRNA Stability/Transcription | 4 | 0 | 0 | 10 | 1 | 0 | 2 | 0 | 3 | 3 | 3 | 3 | 5 | 1 | 3 | 4 | 42 |
| Cell cycle regulation | 0 | 0 | 2 | 0 | 1 | 0 | 0 | 0 | 0 | 0 | 0 | 0 | 0 | 8 | 0 | 0 | 11 |
| Plastids-related expression | 2 | 0 | 0 | 2 | 0 | 0 | 0 | 0 | 5 | 0 | 0 | 5 | 0 | 0 | 0 | 0 | 14 |

**Table S3 –** Presence of nuclear localization signals (NLSs) in the rice SUMOylation machinery proteins. Score values are present for both monopartide and bipartide NLSs by cNLS Mapper.

| **Protein activity** | **Gene/ASF** | **Monopartide** | **Score** | **Bipartide** | **Score** |
| --- | --- | --- | --- | --- | --- |
| SUMO | *OsSUMO1* | - | - | - | - |
|  | *OsSUMO2* | - | - | - | - |
|  | *OsSUMO3* | - | - | - | - |
| E1 SUMO activating enzyme | *OsSAE1.1/2/3* | 1 | 9 | 1 | 5.3 |
|  | *OsSAE2* | 1 (OsSAE2.1/2) | 10.5 | 1 (OsSAE2.1/2/3) | 6.1 |
| E2 SUMO conjugation enzyme | *OsSCE1a.1/2* | - | - | - | - |
|  | *OsSCE1b.1/2* | - | - | - | - |
|  | *OsSCE1c.1/2* | - | - | - | - |
| E3 SUMO ligase | *OsHYP2.1/2* | - | - | - | - |
|  | *OsSIZ1.1/2/3* | - | - | 1 | 6.4 |
|  | *OsSIZ2* | - | - | 1  2 | 5.5  6.1 |
| SUMO protease | *OsELS1.1/2/3* | 1 | 12.5 | 1  2 | 5.5-8.2  5.8 |
|  | *OsSPF1.1/2* | 1 | 7.5-11 | 1  2 | 5  5-5.9 |
|  | *OsOTS3* | 1 | 5.5 | 1 | 5-5.4 |
|  | *OsFUG1* | - | - | 1 (OsUlp1e.1/2/3)  2 (OsUlp1e.1/2/3)  3 (OsUlp1e.1/2) | 5-13.3  5.5  5-7.8 |
|  | *OsELS2* | 1 | 5.5-15 | 1  2  3 | 5.8-8.7  5.1-6  5-5.8 |

**Table S4 –** *In silico* prediction of the subcellular localization of OsELS1 and OsFUG1.

| **Software/Protein** | **OsELS1** | **OsFUG1** |
| --- | --- | --- |
| TargetP | M, RC 5* | Mitochondrial  (M, RC 3*) |
| Predotar | None | Possibly mitochondrial (score 0.26) |

*RC (reliability class): 1 (stronger prediction) to 5 (weakest prediction).

**Table S5 –** Gene locus ID of genes and species used in the phylogenetic analysis. Organisms: *Oryza sativa*, *Arabidopsis thaliana*, *Zea mays*, *Saccharomyces cerevisiae*, *Hordeum vulgare*, *Triticum aestivum*, *Brachypodium distachyon*, *Setaria italica*, *Sorghum bicolor* and *Homo sapiens*.

| **Species/ Machinery** | ***Oryza sativa*** | ***Arabidopsis thaliana*** | ***Zea mays*** | ***Saccharomyces cerevisiae*** | ***Hordeum vulgare*** | ***Triticum aestivum*** | ***Brachypodium distachyon*** | ***Setaria italica*** | ***Sorghum bicolor*** | ***Homo sapiens*** |
| --- | --- | --- | --- | --- | --- | --- | --- | --- | --- | --- |
| **SUMO** | *LOC_Os01g68950* | *AtSUMO1\|At4g26840* | *GRMZM2G082390* | *SMT3* | *MLOC_10713* | *Traes_3DL_397A54CD3* | *Bradi2g58830* | *Si004741m* | *Sb3G402600* | *SUMO1* |
|  | *LOC_Os01g68940* | *AtSUMO2\|At5g55160* | *GRMZM2G053898* |  |  | *TRAES3BF091100240CFD* | *Bradi1g23845* |  | *Sb2G350000* | *SUMO2* |
|  | *LOC_Os07g38690* | *AtSUMO3\|At5g55170* | *GRMZM2G305196* |  |  | *Traes_3AL_D883404F4* | *Bradi1g23832* |  | *Sb2G349800* | *SUMO3* |
|  |  | *AtSUMO5\|At2g3276* |  |  |  | *Traes_3DL_C83285F65* | *Bradi1g23836* |  | *Sb2G350200* | *SUMO4* |
|  |  |  |  |  |  | *Traes_3AL_8EBF5187C* |  |  | *Sb2G349600* |  |
|  |  |  |  |  |  | *Traes_2DS_C57BFD241* |  |  |  |  |
|  |  |  |  |  |  | *Traes_2AS_857FAB255* |  |  |  |  |
| **E1 SUMO Activating Enzyme SAE1** | *LOC_Os11g30410* | *AtSAE1a\|At4g24940* | *GRMZM2G149108* | *Aos1* | *AK250954* | *Traes_3AS_9B09F0AFB* | *Bradi4g18870* | *Si026618m* | *Sb05g120700* | *Q9UBE0\|SAE1* |
|  |  | *AtSAE1b\|At5g50680* |  |  |  |  |  | *Si033137m* |  |  |
| **E1 SUMO Activating Enzyme SAE2** | *LOC_Os07g39780* | *AtSAE2\|At2g21470* | *GRMZM2G129575* | *Uba2* | *MLOC_6744* | *Traes_2DS_E048D56BB* | *Bradi1g23100* | *Si029042m* | *Sb02g037850* | *Q9UBT2\|SAE2* |
|  |  |  |  |  |  | *Traes_2AS_FA3F483D6* |  |  |  |  |
| **E2 SUMO conjugating enzyme SCE** | *LOC_Os10g39120* | *AtSCE1a\|At3g57870* | *GRMZM2G433968* | *UBC9 \|YDL064W* | *MLOC_63571* | *Traes_1BL_A2B322C83* | *Bradi1g77010* | *Si037570m* | *Sb01g030580* | *P63279\|UBC9* |
|  | *LOC_Os04g49130* |  | *GRMZM2G163398* |  | *MLOC_59199* | *Traes_1AL_ED40F32FA* | *Bradi5g19200* | *Si037836m* | *Sb01g049010* |  |
|  | *LOC_Os03g03130* |  | *GRMZM2G06393* |  |  | *Traes_5AL_2618E7CE3* | *Bradi3g32080* | *Si012391m* | *Sb06g026250* |  |
|  |  |  | *GRMZM2G341089* |  |  | *Traes_4BL_F6D28BEBC* |  | *Si011718m* | *Sb06g026270* |  |
|  |  |  | *GRMZM2G038851* |  |  | *Traes_1DL_2086DC17A* |  |  | *Sb06g026280* |  |
|  |  |  | *GRMZM2G070047* |  |  |  |  |  |  |  |
|  |  |  | *GRMZM2G312693* |  |  |  |  |  |  |  |
| **E3 SUMO Ligase HPY2** | *LOC_Os05g48880* | *AtHPY2\|At3g15150* | *GRMZM2G022065* | *MMS21\|YEL019C* | *MLOC_48639* | *Traes_1AL_324513CB7* | *Bradi2g16600* | *Si022832m* | *Sb09g231900* | *Q96MF7\|NSE2* |
|  |  |  |  |  |  |  | *Bradi2g16585* |  |  |  |
| **E3 SUMO Ligase SIZ** | *LOC_ Os05g03430* | *AtSIZ1\|At5g60410* | *GRMZM2G155123* | *YDR409W\|SIZ1* | *MLOC_66261* | *Traes\|1BS_5CA2068F7* | *Bradi2g38030* | *Si021176m* | *Sb09g026500* | *O75925\|PIAS1* |
|  | *LOC_Os03g50980* |  | *GRMZM2G337659* | *YOR156C\|SIZ2* | *MLOC_72995* | *TRAES3BF024600030CFD* | *Bradi2g62697* | *Si021195m* | *Sb08g002300* | *Q9Y6X2\|PIAS3* |
|  |  |  |  |  |  |  | *Bradi4g45080* | *Si021164m* |  | *O00257\|CBX4* |
|  |  |  |  |  |  |  | *Bradi4g26720* | *Si021166m* |  | *P49792\|RBP2* |
|  |  |  |  |  |  |  |  | *Si009415m* |  | *O75928\|PIAS2* |
|  |  |  |  |  |  |  |  | *Si026017m* |  | *Q8IY92\|SLX4* |
| **SUMO Protease** | *LOC_Os03g22400* | *AtESD4\|At4g15880* | *GRMZM5G849959* | *ULP1* | *MLOC_69186* | *Traes_4AS_A10921326* | *Bradi1g62760* | *Si035922m* | *Sb01g375300* | *SENP1* |
|  | *LOC_Os01g25370* | *AtELS2\|At4g00690* | *GRMZM2G012601* | *ULP2* | *MLOC_3742* | *Traes_3AS_0E7CB4B4A* | *Bradi1g18100* | *Si029656m* | *Sb02g413600* | *SENP2* |
|  | *LOC_Os05g11770* | *AtUELS1\|At3g06910* | *GRMZM2G010505* |  | *MLOC_81989* | *Traes_3DS_5A21E47BA* | *Bradi2g12720* | *Si001154m* | *Sb03g160000* | *SENP3* |
|  | *LOC_Os03g29630* | *AtOTS2\|At1g10570* | *GRMZM2G088653* |  | *MLOC_71322* | *TRAES3BF090600010CFD* | *Bradi3g60530* | *Si001568m* | *Sb03g360400* | *SENP5* |
|  | *LOC_Os01g53630* | *AtOTS1\|At1g60220* |  |  | *MLOC_67565* | *Traes_5BL_DE7669EED* |  | *Si033994m* | *Sb03g289700* | *SENP6* |
|  |  | *AtFUG1\|At3g48480* |  |  | *MLOC_74807* |  |  |  |  | *SENP7* |
|  |  | *AtSPF2\|At4g33620* |  |  |  |  |  |  |  |  |
|  |  | *AtSPF1\|At1g09730* |  |  |  |  |  |  |  |  |

**Table S6 –** List of gene/transcript primers used for the genotyping the T-DNA insertion lines.

| **Gene Locus** | **Primer ID** | **Primer Sequence (5'-3')** |
| --- | --- | --- |
| Os03g03130 | OsSCE1a-F1 | AGACTCCTCCTAGCTGGTCTCGTAG |
|  | OsSCE1a-R1 | TCCTCCTCGATCAACTTCAACTTT |
| Os04g49130 | OsSCE1c-F1 | CGAGGTAAAAGGAACGGAAATG |
|  | OsSCE1c-R1 | TTAAGCAACCAACGGAGCA |
| Os05g03430 | OsSIZ1-F1 | GATATACTGAAAGGTACCAG |
|  | OsSIZ1-R1 | TCACCAAAGGTTAGCAGCAAC |
| Os01g25370 | OsELS1-F1 | TGCAGTGCTTGAACGAAAC |
|  | OsELS1-R1 | AGACACAAACCAAGCAACTA |
| Os03g22400 | OsFUG1-F1 | GGGTAAACCTAGGCACGACGA |
|  | OsFUG1-R1 | AAACAACATGCAGCAGGGTC |
|  | Hygromycin-F1 | AATAGCTGCGCCGATGGTTTCTACA |
|  | Hygromycin-R1 | AACATCGCCTCGCTCCAGTCAATG |
|  | pLeftB-2715 | ACGTCCGCAATGTGTTATTAA |
|  | pLeftB-2707 | GGTGAATGGCATCGTTTGAA |
|  | pLeftB-2772 | TCCGAAACTATCAGTGTCTAGCT |
|  | pRightB | AACGCTGATCAATTCCACAG |
|  | LB-RMD | AATCCAGATCCCCCGAAT |

**Table S7 –** List of gene/transcript primers used in Real-time qPCR analysis.

| **Gene Locus** | **Gene/ASF** | **Forward Primer (5'-3')** | | **Reverse Primer (5'-3')** | |
| --- | --- | --- | --- | --- | --- |
| Os04g49130 | *OsSCE1c.1* | qRT-15F1 | GATGATCCAAACCCTAACTCT | qRT-15R1 | TGTACTCCTCCATGTCCTTC |
|  | *OsSCE1c.2* | qRT-15F | CCGCATGGAAACCTTCAATTA | qRT-15R2 | CATGTCATAACTAAACCTTCCT |
| Os05g03430 | *OsSIZ1.1-2* | qRT-16F | GCAAGGTCAAAGGCATCATG | qRT-16R1 | CTGCTGTATGTATCATCAACAA |
|  | *OsSIZ1.3* | qRT-16F3 | CATTAGCAGGAAAATGCAAATC | qRT-16R3 | ATCGCTGATCTTCACACTGG |
| Os05g48880 | *OsHPY2.1* | qRT-17F1 | CTAATCAGATGACGGATTTCG | qRT-17R1 | GAACTGCCGAATGAGCAGAT |
|  | *OsHPY2.2* | qRT-17F2.1 | GCGCTGGAACTGGTTGCTT | qRT-17R2 | GTCCGCACCTGATTAGATG |
| Os01g25370 | *OsELS1.1* | qRT-19F1 | GGTGGGATTGTGGCATGTT | qRT-19R1 | AGTAATGCATATGTTTCTGTCC |
|  | *Os ELS1.2* | qRT-19F2.1 | CAAGGTATCTTGTAGATGAGG | qRT-19R2 | GAGAAAAATACCCATTCTCTTG |
|  | *Os ELS1.3* | qRT-19F3.1 | GCAGTCATAAACATAAGGGAC | qRT-19R3 | CTACTTACTAAAGTTCTCAATG |
| Os03g22400 | *Os FUG1.1* | qRT-20F1.2 | CTTGATTCTCTTGGTTGTGTG | qRT-20R1 | CAATATATCTAGCCAGCACTC |
|  | *Os FUG1.2* | qRT-20F2.1 | ACTCACAGGCTAGATATATTGC | qRT-20R2.1 | ACAGTCCCACCCATTTTGC |
|  | *OsFUG1.3* | qRT-20F3.3 | TGCAATGAACTTATGAGTGTTC | qRT-20R3.3 | ACCTCCTGAGGGAAAAACTC |
| Os05g11770 | *OsSPF1.1* | qRT-21F1.3 | ACTCACTCGGAATTCTCTCT | qRT-21R1.3 | GCTTCCGAGAAGGCACG |
|  | *Os SPF1.2* | qRT-21F2.2 | GTGTAATTTCAGATTTAGCTGA | qRT-21R2.2 | ACCATTTCACCCAAAACATTG |
| Os03g29630 | *OsELS2* | qRT-22F | CACCAGTTCATGGCTGAAAA | qRT-22R | GGTAGGTCATATGTAGCATG |
| Os01g53630 | *OsOTS3* | qRT-23F2 | GCTGCAGGAGTCGATCAAG | qRT-23R | GGCCTCCAAGTTATGACACC |
| Os03g50980 | *OsSIZ2* | qRT-24F | GCAAGGAAGGAAACAGGAACT | qRT-24R | TTTTTGTTTGGCAAGCCATT |
| Os03g03130 | *OsSCE1a.1* | qRT-25F1 | CACCCACACGGTTTCGTC | qRT-25R1 | CTTGCTTGCCGGGGATTGT |
|  | *OsSCE1a.2* | qRT-25F2.2 | GTGCCTCTCGATCTCAACG | qRT-25R2.2 | CTACGGAACCAAATCAGAAC |
| Os10g39120 | *OsSCE1b.1* | qRT-26F1 | ACGGGTTCGTGGCGAAGC | qRT-26R | CTTCCCAATCGGTCCCCTG |
| Os07g39780 | *OsSAE2.1* | qRT-27F1.3 | TTATTTGGGGTGCAGGCGG | qRT-27R1 | GATGTGAATATCGCGGAATC |
|  | *OsSAE2.2* | qRT-27F2 | CTGAACGGCACCACACCAC | qRT-27R2 | CGAGGTGACGGCTTTGATG |
|  | *OsSAE2.3* | qRT-27F3.1 | GGCACTTCATTGGCTAACAAT | qRT-27R3.1 | CAAATGGCTCGATAGGCATC |
| Os11g30410 | *OsSAE1.1* | qRT-28F1 | GTGTGATAAAATGTCTTTAAGTG | qRT-28R | ATGCCGCCAAGGATTGCAC |
|  | *OsSAE1.2* | qRT-28F2.1 | GGTGTGGTAAATAAACTGCAG | qRT-27R2.1 | TACAGGAGGATGTTGCTTCT |
|  | *OsSAE1.3* | qRT-28F3 | GTGTGATAAAATGTGAGTCTCA | qRT-28R | ATGCCGCCAAGGATTGCAC |
| Os01g6895/40 | *OsSUMO1/2* | qRT-29F | TCCGCATCAAGAGATCTACGC | qRT-29R | CGGAGCCTACGACCATCAAAT |
| Os07g38690 | *OsSUMO3* | qRT-30F | GGAGAGTACGTCACGCTGAA | qRT-30R | GCGCGGTCGTAGTAGAAGTC |
| Os02g42314 | *OsUBC2* | qRT-OsUBC2qF | TTGCATTCTCTATTCCTGAGCA | qRT-OsUBC2qR | CAGGCAAATCTCACCTGTCTT |
| Os03g08020 | *OseEF-1a* | qRT-eEF-1a-F2 | TGGTGACCAAGATCGACAGA | qRT-eEF-1a-R2 | GCATCACCGTTCTTGAGGA |
| Os05g08980 | *OsEP* | OsEP_qPCRa_Fw | TGAGCAAAATGGTGGAAAGC | OsEP_qPCRa_Rv | CAGTTGCAACCCCTGTATGA |

**Table S8 –** Summary of the statistical analysis of rice SUMOylation machinery genes in shoots (Sh) and roots (Rt), in normal growth conditions**.**

| **Family** | **SUMOylation machinery gene (tissue)** | **Statistical significance** | **Family** | **SUMOylation machinery gene (tissue)** | **Statistical significance** |
| --- | --- | --- | --- | --- | --- |
| **SUMO** | *OsSUMO1-2*(Sh) vs *OsSUMO3*(Sh) | ** | **SUMO Proteases** | *OsFUG1.1*(Sh) vs *Os FUG1.2*(Sh) | ns |
|  | *OsSUMO1-2*(Sh) vs *OsSUMO1-2*(Rt) | ns |  | *Os FUG1.1*(Sh) vs *Os FUG1.3*(Sh) | ns |
|  | *OsSUMO3*(Sh) vs *OsSUMO3*(Rt) | ns |  | *Os FUG1.1*(Sh) vs *OsELS1.1*(Sh) | *** |
|  | *OsSUMO1-2*(Rt) vs *OsSUMO3*(Rt) | * |  | *Os FUG1.1*(Sh) vs *OsSPF1.1*(Sh) | ns |
| **E1** | *OsSAE1.1*(Sh) vs *OsSAE1.2*(Sh) | *** |  | *Os FUG1.1*(Sh) vs *OsELS2*(Sh) | ns |
|  | *OsSAE1.1*(Sh) vs *OsSAE1.3*(Sh) | *** |  | *Os FUG1.1*(Sh) vs *OsOTS3*(Sh) | ns |
|  | *OsSAE1.1*(Sh) vs *OsSAE2.2*(Sh) | *** |  | *Os FUG1.1*(Sh) vs *Os FUG1.1*(Rt) | ns |
|  | *OsSAE1.1*(Sh) vs *OsSAE1.1*(Rt) | ns |  | *Os FUG1.2*(Sh) vs *Os FUG1.3*(Sh) | *** |
|  | *OsSAE1.2*(Sh) vs *OsSAE1.3*(Sh) | ns |  | *Os FUG1.2*(Sh) vs *Os FUG1.2*(Rt) | ns |
|  | *OsSAE1.2*(Sh) vs *OsSAE1.2*(Rt) | ns |  | *Os FUG1.3*(Sh) vs *Os FUG1.3*(Rt) | ns |
|  | *OsSAE1.3*(Sh) vs *OsSAE1.3*(Rt) | ns |  | *OsELS1.1*(Sh) vs *Os ELS1.2*(Sh) | *** |
|  | *OsSAE2.1*(Sh) vs *OsSAE2.2*(Sh) | ns |  | *Os ELS1.1*(Sh) vs *Os ELS1.3*(Sh) | *** |
|  | *OsSAE2.1*(Sh) vs *OsSAE2.3*(Sh) | ns |  | *Os ELS1.1*(Sh) vs *OsSPF1.1*(Sh) | *** |
|  | *OsSAE2.1*(Sh) vs *OsSAE2.1*(Rt) | ns |  | *Os ELS1.1*(Sh) vs *OsELS2*(Sh) | *** |
|  | *OsSAE2.2*(Sh) vs *OsSAE2.3*(Sh) | ns |  | *Os ELS1.1*(Sh) vs *OsOTS3*(Sh) | *** |
|  | *OsSAE2.2*(Sh) vs *OsSAE2.2*(Rt) | ns |  | *Os ELS1.1*(Sh) vs *Os ELS1.1*(Rt) | *** |
|  | *OsSAE2.3*(Sh) vs *OsSAE2.3*(Rt) | ns |  | *Os ELS1.2*(Sh) vs *Os ELS1.3*(Sh) | ns |
|  | *OsSAE1.1*(Rt) vs *OsSAE1.2*(Rt) | *** |  | *Os ELS1.2*(Sh) vs *Os ELS1.2*(Rt) | ns |
|  | *OsSAE1.1*(Rt) vs *OsSAE1.3*(Rt) | *** |  | *Os ELS1.3*(Sh) vs *Os ELS1.3*(Rt) | ns |
|  | OsSAE1.1(Rt) vs OsSAE2.2(Rt) | *** |  | *OsSPF1.1*(Sh) vs *Os SPF1.2*(Sh) | ns |
|  | *OsSAE1.2*(Rt) vs *OsSAE1.3*(Rt) | ns |  | *Os SPF1.1*(Sh) vs *OsELS2*(Sh) | ns |
|  | *OsSAE2.1*(Rt) vs *OsSAE2.2*(Rt) | ns |  | *Os SPF1.1*(Sh) vs *OsOTS3*(Sh) | ns |
|  | *OsSAE2.1*(Rt) vs *OsSAE2.3*(Rt) | ns |  | *Os SPF1.1*(Sh) vs *Os SPF1.1*(Rt) | ns |
|  | *OsSAE2.2*(Rt) vs *OsSAE2.3*(Rt) | ns |  | *Os SPF1.2*(Sh) vs *Os SPF1.2*(Rt) | ns |
| **E2** | *OsSCE1a.1*(Sh) vs *OsSCE1a.2*(Sh) | *** |  | *OsELS2*(Sh) vs *OsOTS3*(Sh) | ns |
|  | *OsSCE1a.1*(Sh) vs *OsSCE1b.1*(Sh) | *** |  | *OsELS2*(Sh) vs *OsELS2*(Rt) | ns |
|  | *OsSCE1a.1*(Sh) vs *OsSCE1c.1*(Sh) | *** |  | *OsOTS3*(Sh) vs *OsOTS3*(Rt) | ns |
|  | *OsSCE1a.1*(Sh) vs *OsSCE1c.2*(Sh) | *** |  | *OsFUG1.1*(Rt) vs *Os FUG1.2*(Rt) | ns |
|  | *OsSCE1a.1*(Sh) vs *OsSCE1a.1*(Rt) | ns |  | *Os FUG1.1*(Rt) vs *Os FUG1.3*(Rt) | ns |
|  | *OsSCE1a.2*(Sh) vs *OsSCE1b.1*(Sh) | *** |  | *Os FUG1.1*(Rt) vs *OsELS1.1*(Rt) | *** |
|  | *OsSCE1a.2*(Sh) vs *OsSCE1c.1*(Sh) | ns |  | *Os FUG1.1*(Rt) vs *OsSPF1.1*(Rt) | ns |
|  | *OsSCE1a.2*(Sh) vs *OsSCE1c.2*(Sh) | ns |  | *Os FUG1.1*(Rt) vs *OsELS2*(Rt) | ns |
|  | *OsSCE1a.2*(Sh) vs *OsSCE1a.2*(Rt) | ns |  | *Os FUG1.1*(Rt) vs *OsOTS3*(Rt) | ns |
|  | *OsSCE1b.1*(Sh) vs *OsSCE1c.1*(Sh) | *** |  | *Os FUG1.2*(Rt) vs *Os FUG1.3*(Rt) | ns |
|  | *OsSCE1b.1*(Sh) vs *OsSCE1c.2*(Sh) | *** |  | *OsELS1.1*(Rt) vs *OsELS1.2*(Rt) | *** |
|  | *OsSCE1b.1*(Sh) vs *OsSCE1b.1*(Rt) | ns |  | *Os ELS1.1*(Rt) vs *OsELS1.3*(Rt) | *** |
|  | *OsSCE1c.1*(Sh) vs *OsSCE1c.2*(Sh) | ns |  | *Os ELS1.1*(Rt) vs *OsSPF1.1*(Rt) | *** |
|  | *OsSCE1c.1*(Sh) vs *OsSCE1c.1*(Rt) | ns |  | *Os ELS1.1*(Rt) vs *OsELS2*(Rt) | *** |
|  | *OsSCE1c.2*(Sh) vs *OsSCE1c.2*(Rt) | ns |  | *OsELS1.1*(Rt) vs *OsOTS3*(Rt) | *** |
|  | *OsSCE1a.1*(Rt) vs *OsSCE1a.2*(Rt) | *** |  | *OsELS1.2*(Rt) vs *OsELS1.3*(Rt) | ns |
|  | *OsSCE1a.1*(Rt) vs *OsSCE1b.1*(Rt) | *** |  | *OsELS1.3*(Rt) vs *OsELS2*(Rt) | ns |
|  | *OsSCE1a.1*(Rt) vs *OsSCE1c.1*(Rt) | *** |  | *OsELS1.3*(Rt) vs *OsOTS3*(Rt) | ns |
|  | *OsSCE1a.1*(Rt) vs *OsSCE1c.2*(Rt) | *** |  | *OsSPF1.1*(Rt) vs *OsSPF1.2*(Rt) | ns |
|  | *OsSCE1a.2*(Rt) vs *OsSCE1b.1*(Rt) | *** |  | *OsSPF1.1*(Rt) vs *OsELS2*(Rt) | ns |
|  | *OsSCE1a.2*(Rt) vs *OsSCE1c.1*(Rt) | ns |  | *OsSPF1.1*(Rt) vs *OsOTS3*(Rt) | ns |
|  | *OsSCE1a.2*(Rt) vs *OsSCE1c.2*(Rt) | ns |  | *OsELS2*(Rt) vs *OsOTS3*(Rt) | ns |
|  | *OsSCE1b.1*(Rt) vs *OsSCE1c.1*(Rt) | *** |  |  |  |
|  | *OsSCE1b.1*(Rt) vs *OsSCE1c.2*(Rt) | *** |  |  |  |
|  | *OsSCE1c.1*(Rt) vs *OsSCE1c.2*(Rt) | ns |  |  |  |
| **E3** | *OsSIZ1.1-2*(Sh) vs *OsSIZ1.3*(Sh) | *** |  |  |  |
|  | *OsSIZ1.1-2*(Sh) vs *OsSIZ2*(Sh) | *** |  |  |  |
|  | *OsSIZ1.1-2*(Sh) vs *OsSIZ1.1-2*(Rt) | ** |  |  |  |
|  | *OsSIZ1.3*(Sh) vs *OsSIZ2*(Sh) | ns |  |  |  |
|  | *OsSIZ1.3*(Sh) vs *OsSIZ1.3*(Rt) | ns |  |  |  |
|  | *OsSIZ2*(Sh) vs *OsSIZ2*(Rt) | ns |  |  |  |
|  | *OsSIZ1.1-2*(Rt) vs *OsSIZ1.3*(Rt) | *** |  |  |  |
|  | *OsSIZ1.1-2*(Rt) vs *OsSIZ2*(Rt) | *** |  |  |  |
|  | *OsSIZ1.3*(Rt) vs *OsSIZ2*(Rt) | ns |  |  |  |
|  | *OsHPY2.1*(Sh) vs *OsHPY2.2*(Sh) | ns |  |  |  |
|  | *OsHPY2.1*(Sh) vs *OsHPY2.1*(Rt) | * |  |  |  |
|  | *OsHPY2.2*(Sh) vs *OsHPY2.2*(Rt) | ns |  |  |  |
|  | *OsHPY2.1*(Rt) vs *OsHPY2.2*(Rt) | ** |  |  |  |

ns – not significant, * - p<0.05, ** - p<0.01, *** - p<0.001

**Supplemental Figures**


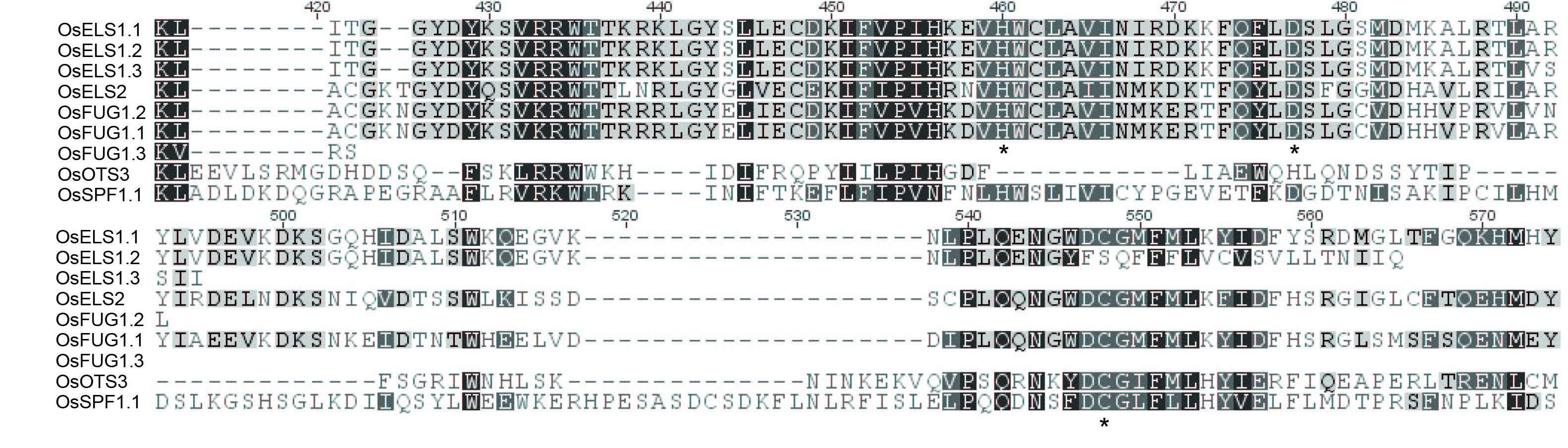


**Figure S1 –** Alignment of the C-terminal region of the studied rice SUMO proteases. The catalytic triad is highlighted with an asterisk “*”.


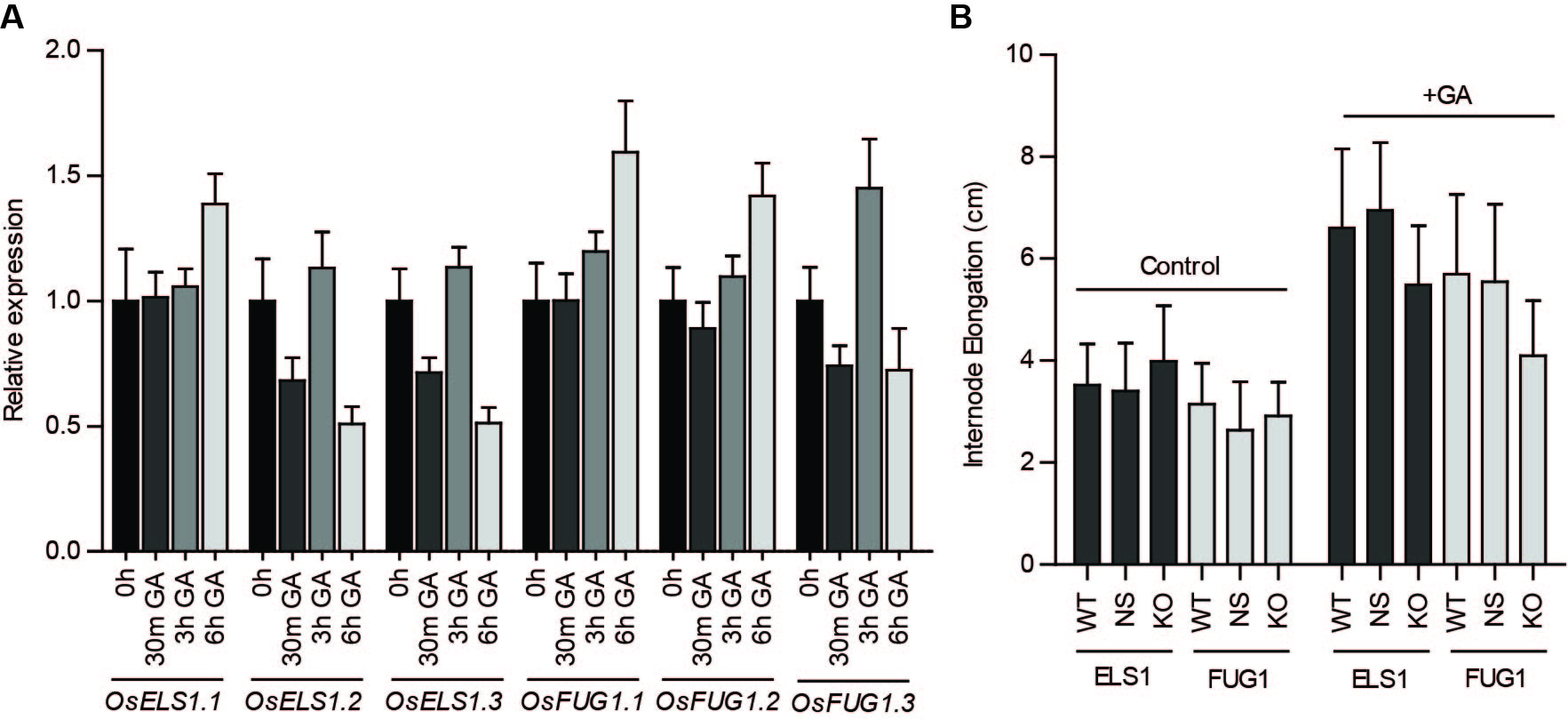


**Figure S2** – (A) *OsELS1* and *OsFUG1* and respective ASFs transcriptional behavior in response to 30 min, 3h and 6h of 100 µM of GA. Data was obtained from shoot samples of 8-day-old rice seedlings by qPCR. (B) Internode elongation (measured in cm) of seedlings subjected to 100 µM GA for 3 days at the 12-day old stage. We used the T-DNA insertion lines of *OsELS1* and *OsFUG1*, respective wild types and negative segregant rice seedlings. A Bonferroni’s Multiple Comparison Test for the GA response data was performed (p< 0.05) and showed all data not significantly different.


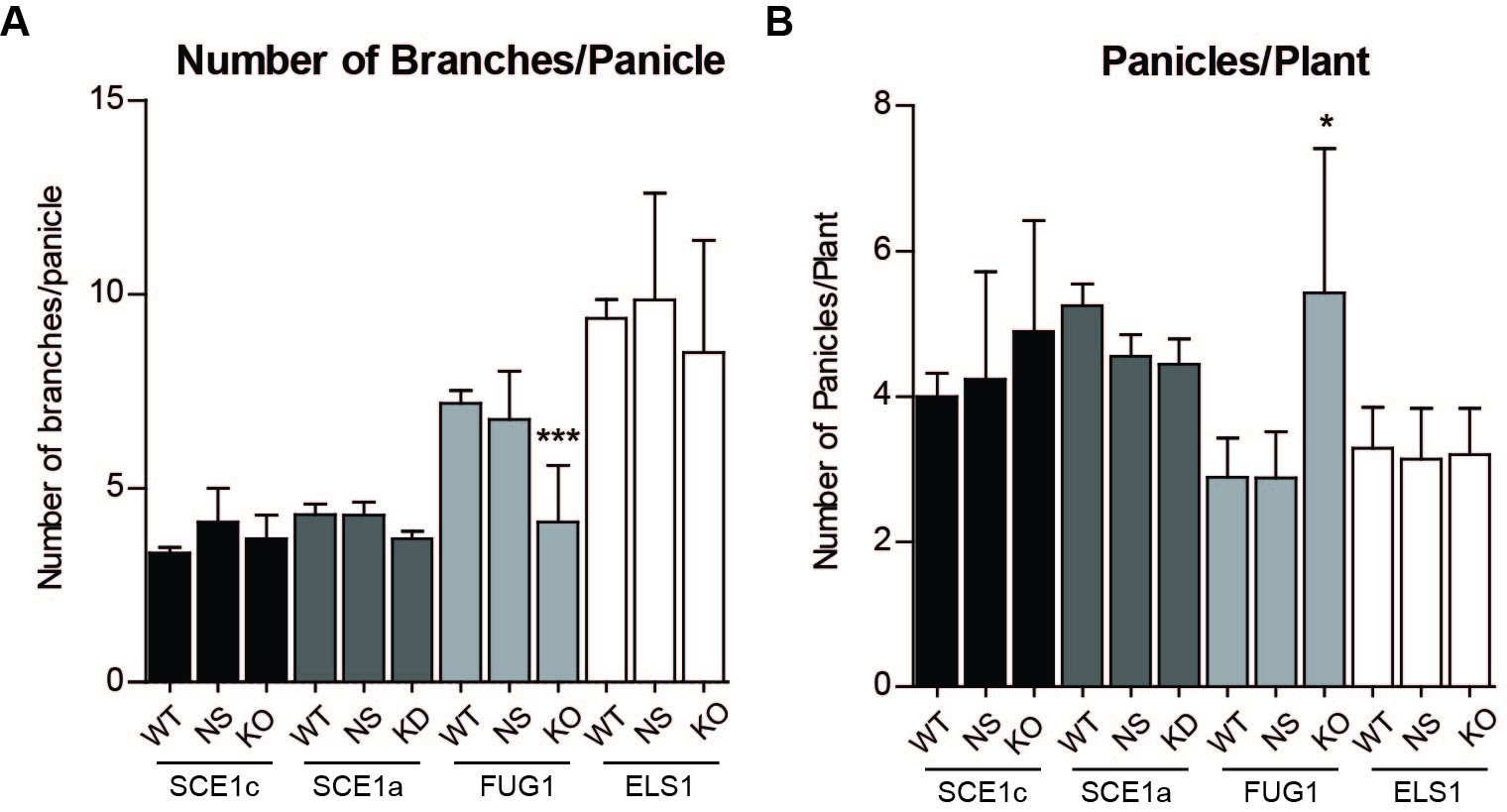


**Figure S3 –** Phenotype of the T-DNA insertion lines. (A) Number of branches per panicle and (B) number of panicles per plant. Asterisks represent statistical significance (*p-value* < 0.05). Only the significant differences between the T-DNA lines and their respective wild type/negative segregant lines are depicted.
